# A Decade of Progress on MAO-Treated Tantalum Surfaces: Advances and Contributions for Biomedical Applications

**DOI:** 10.3390/nano12142319

**Published:** 2022-07-06

**Authors:** Luísa Fialho, Cristiana F. Almeida Alves, Sandra Carvalho

**Affiliations:** 1 CEMMPRE Mechanical Engineering Department, University of Coimbra, 3030-788 Coimbra, Portugal; alvescristiana89@gmail.com; 2 INL—International Iberian Nanotechnology Laboratory, Av. Mestre José Veiga s/n, 4715-330 Braga, Portugal; 3 IPN—LED&MAT—Instituto Pedro Nunes, Laboratório de Ensaios, Desgaste e Materiais, Rua Pedro Nunes, 3030-199 Coimbra, Portugal

**Keywords:** bioactivity, biocompatibility, functionalization, micro-arc oxidation, tantalum

## Abstract

Micro-structured coatings with functional properties have been investigated due to a wide range of applications. It is known that micro-structures can play an important role in surface interactions determining the materials’ performance. Amongst the other materials, there has been an increasing interest in tantalum oxide (Ta_2_O_5_). This attention is mainly due to its variety of properties: biocompatibility and bioactivity; high dielectric constant; good thermal and chemical stability; excellent corrosion and mechanical resistance. Moreover, there is a wide range of applications in which the properties can be fitted. Furthermore, according to the final application, these properties can be enhanced or tailored through surface micro-structures manipulation. Due to this purpose, over the past decade, Ta surface modification by micro-arc oxidation (MAO) has been investigated mostly for biomedical applications. Therefore, this review focuses on Ta surface functionalization using the MAO technique. A clear understanding of the micro-discharge phenomena and the formation mechanism of a Ta_2_O_5_ anodic coating by MAO is supplied. The Ta_2_O_5_ coating morphology, topography, chemistry, and structure are explored, establishing their correlation with the MAO parameters. Additionally, an understanding of Ta_2_O_5_’s biological, mechanical, and electrochemical properties is provided and reviewed.

## 1. Introduction

Tantalum oxide (Ta_2_O_5_) micro-structured coatings present interesting properties that make them suitable for a large range of applications. These coatings are used for storage capacitors and resistors in electronic [1,2] and microelectronic industries [1,3,4] because they show a high dielectric strength, high melting point and thermal stability [5], excellent corrosion [6,7,8,9], and abrasion resistance [10], which make them also a reliable option for biomedical instruments [6,7,11,12,13,14] and chemical equipment (e.g., optical devices) [15]. They are biocompatible [16,17,18,19] too, being a good option for bioengineering implants application [6,11,20,21,22,23,24]. Ta_2_O_5_ coatings also have a wide bandgap, exhibiting a high photocatalytic activity [25]. Furthermore, Ta-based materials (nitrides, oxynitrides) have been suggested for photochemical [25,26,27,28] and metal-oxide-semiconductor (MOS) transistors [29], as well as SiO-Ta_2_O_5_ films for memory and optical devices [30,31,32].

Micro-structured Ta_2_O_5_ film can be produced by a wide number of techniques, including methods such as chemical solution deposition, such as sol–gel (e.g., [4]) and polymer-assisted deposition (e.g., [6,20]); physical vapor deposition, such as magnetron sputtering (e.g., [21,33,34,35]) and pulsed laser deposition (e.g., [3]); chemical vapor deposition (e.g., [13,22]); electrochemical-processes-anodization (e.g., [25,32,36,37,38,39,40,41,42,43]); and micro-arc oxidation (e.g., [23]). One of the most common electrochemical processes used for Ta_2_O_5_ film production is anodization, which is a low-cost and industrialized technique, although it uses environmentally harmful chemicals [44,45]. Micro-arc oxidation (MAO) is a promising emerging technique for surface modification that offers an alternative track for the production of well-adhered oxide coatings using weak alkaline electrolytes instead of the highly acidic electrolytes associated with the anodizing process. The main difference between MAO and anodization is that MAO works at higher voltages above the Ta_2_O_5_ breakdown potential (around 200 V [44]). This review paper explores the complexity of the MAO process onto Ta surfaces and the structures’ growth mechanism from relatively compact to porous, passing from nodular and dendric morphologies, and dissects the attained surface properties regarding the MAO parameters, providing the opportunity to be used in a range of applications, particularly in biomedical.

## 2. Understanding the Principles of the MAO Process onto Ta Surfaces

Derived from the anodization process, the MAO technique, also known as plasma electrolytic oxidation (PEO), is distinguished since it works with a higher voltage [45,46,47,48]: when the applied voltage is high enough (higher than the breakdown potential of the oxide layer), a dielectric breakdown occurs through the thickness of the growing oxide coatings [49,50]. The MAO step-up and procedure are relatively similar to conventional anodization, although oxidation occurs when there is a combination of metal and oxygen atoms or ions within the discharge plasma (as it cools and collapses) instead of a continuous transport of ions through the electrolyte and within a thin oxide coating [45].

This process allows for the production of thick oxide coatings, which often result in a harder and crystalline structure due to the large amounts of heat released by the discharges [45,46]. The maximum thickness is not dependent on the applied voltage but instead is reached at stable discharges [45]. Additionally, the MAO process allows for the formation of durable and well-adherent coatings on many metals (e.g., Ti, Mg, Zr, and Ta) [23,45,49], as well as for the creation of surfaces with a wide range of colors and textures [45]. In addition, the resulting oxide coating can have relatively high levels of porosity [45,48], and it can be enriched with components from the electrolyte [23,51]. The MAO oxide coating can benefit from mechanical stability, such as a reduced stiffness and elastic’s modulus, conferring sliding, corrosion, and fatigue resistance. MAO enables the development of functional surfaces for biomedical applications. This process provides the formation of porous structures, raising the specific surface area, which allows a strong bonding to the bone [52]. Furthermore, the incorporation of species from the electrolyte, such as calcium (Ca) and phosphorous (P), enhances bioactivity [45,49]. These surface features can be tailored by the applied voltage, anodic current density, and electrolyte composition and concentration [23,52]. It is important to highlight that the MAO process, like anodization, is industrially well-accepted.

### 2.1. Transient Plasma Discharge Mechanism

**Figure 1:**
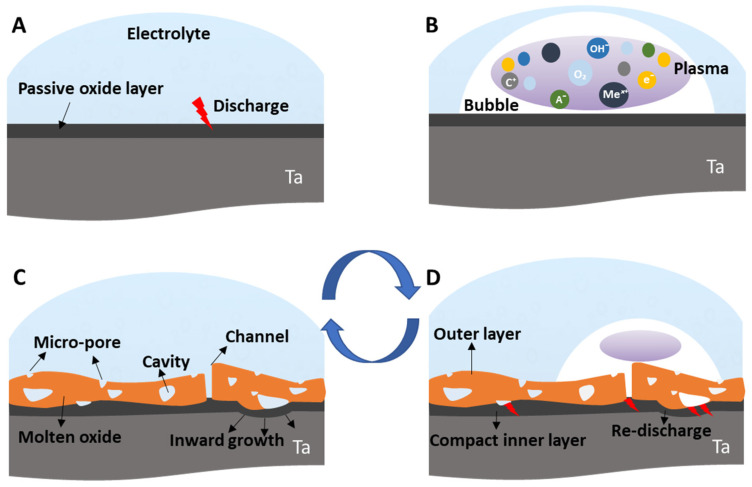
Schematic illustration of the representative growth mechanism of the Ta_2_O_5_ coating by MAO: (**A**) start of the spark discharges after the rapid formation of the oxide layer; (**B**) formation of the bubble and ignition of the plasma; (**C**) new oxide formation; (**D**) start of the new discharges with a generation of a gas bubble and plasma, leading to the formation of the new oxide coating.

At the initial stage of the MAO process, a large number (cascade) of micro-discharges distributed over the surface are formed under high voltages [45], consequently initiating a plasma formation, leading to a series of complex electrochemical, plasma, and thermo-chemical reactions under high temperature and pressure (Figure 1) [23,53,54].

The electric field passing through the oxide (passive layer bandgap) increases, and if the applied voltage reaches the breakdown level, i.e., the dielectric strength of the oxide, the discharge occurs (Figure 1A) [45,55]. This discharge is initiated by the electron flow from the electrolyte to the substrate, crossing through the passive layer, initially in particular areas (the micro-defects). At the moment of a discharge, a gas bubble is formed, which rapidly expands due to the heat flow, generating plasma formation and growth [45,53]. The resultant plasma contains species from the substrate (metallic cations—Me^x+^), the electrolyte (cations—C^+^, and anions—A^−^, OH^−^), and the growing oxide coating, as well as O_2_ gas, which results from the ionization of water molecules (Figure 1B). These O_2_ molecules can combine with metallic cations from the substrate in the plasma. In the discharge channel, the alloy elements (from the substrate and existing oxide) are molten, and consequently oxidized as a result of the elevated temperature, and are transported from inside the channel to the substrate [56]. When the electric resistance rises, the discharge stops, the bubble shrinks [45,52,57], the plasma cools and collapses, and the reaction products, as well as the erupted molten oxide, are deposited on the surface forming the new oxide (Figure 1C) [56]. The new oxide condenses and is quickly redistributed within the layer structure. Then, the coating solidifies, and a disordered microstructure architecture is created (Figure 1C) [45,52,57].

This process runs endlessly, the substrate is consumed at the site of the discharge, and the oxide is gradually formed in the vicinity [45], growing simultaneously inward and outward from the original metallic surface [47], with a continuous reconstruction throughout the oxide thickness (Figure 1D). Moreover, several phenomena take place during the MAO process for metallic oxidation: the melting and vaporization of the metallic substrate, the melting of the oxide coating, the initiation and sustainment of the plasma, the water vaporization, the electrical heating of the electrolyte, and, finally, the metal-to-oxide conversion [45]. The developed oxide is broadly composed of two layers: a porous outer layer and a compact inner layer (Figure 1D) [47]. As the oxide thickness increases, a tendency for the discharge to become more energetic and more dispersed in time and space [45,57,58,59] is observed, which increases the duration of the process [46]. In addition, oxygen liberation at the anode occurs at a high anodic current due to the oxidation of water or oxide ions [47]. The lifetime of a discharge cascade is terminated when the oxide reaches enough thickness in that specific site, presenting a higher electrical resistance than in the vicinity.

**Figure 2:**
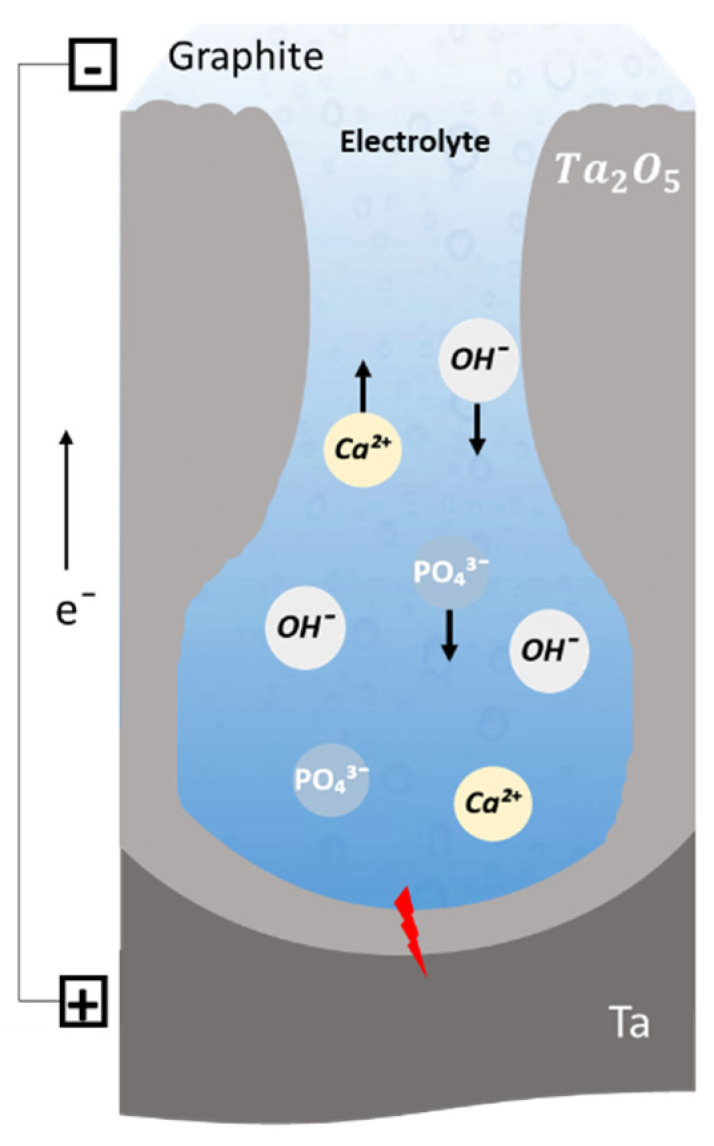
Schematic illustration of transport phenomena that takes place during the MAO process.

During MAO, with anodic polarization, the electron flow through the oxide coating is limited by the relatively slow rate of the OH^−^ ions’ arrival through the electrolyte under the influence of the applied potential (Figure 2). Like in anodization, electrolyte composition, which is even aqueous and alkaline, influences the discharge ignition since it must be conductive, and may influence the chemical reactions of ionized species, as well as the metallic substrate oxidation [45,60].

**Figure 3:**
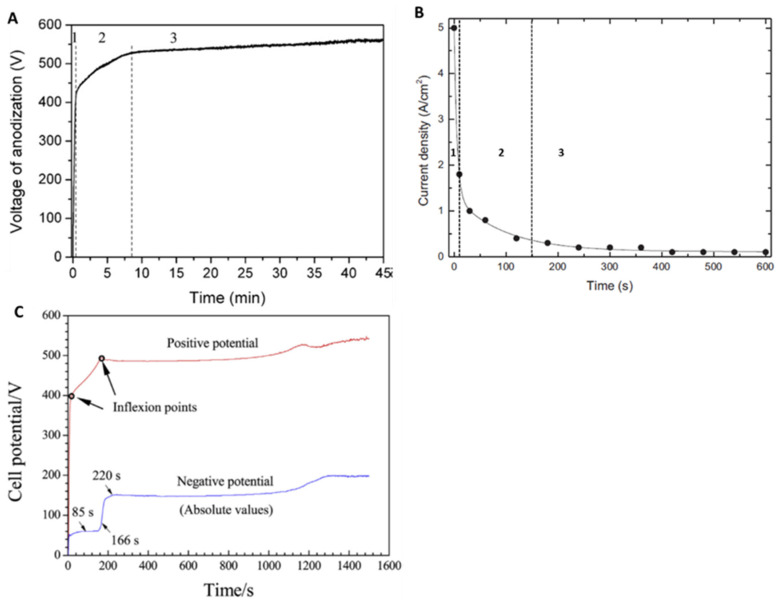
(**A**) Voltage–time curve. Adapted with permission from Ref. [46]. Copyright 2011 Elsevier. (**B**) Current–density curve. Reprinted with permission from Ref. [55]. Copyright 2019 Elsevier. (**C**) Cell-potential–time responses recorded during MAO treatment. Adapted with permission from Ref. [63]. Copyright 2020 Elsevier.

Using the analysis of the voltage/current density–-time curve, the MAO process, carried out in DC mode onto a Ta surface, can be divided into three distinct stages as displayed in Figure 3A,B [46,58,61,62]. In the first seconds of a galvanostatic mode (constant current) (Figure 3A), the voltage sharply increases in a short time, which is defined as the first stage of the MAO process. Like traditional anodization, a compact oxide coating is formed in this stage. Throughout the anodic coating growth, the electric field strength for a given current density remains constant during the anodic growth, and the ionic current is two or three orders of magnitude larger than the electronic component. Furthermore, the voltage increases with the increase in the anodic coating thickness to maintain the electric field strength constant [45,58,61]. In this stage, galvanoluminescence is not observed. During the electrolytic oxidation, electrons are injected into the conduction band of the anodic layer and are accelerated by the electric field producing avalanches using an ionization mechanism [61]. When the electronic current avalanche reaches a critical value (the breakdown potential of the oxide layer), dielectric breakdown occurs, which is reflected by an apparent deflection from the linearity of the V-t curve and the appearance of small sparks. Hence, the uniform coating thickening is terminated and starts a so-called breakdown voltage (second stage) [45,58,61] that activates and induces a fast increase in galvanoluminescence intensity [61]. The breakdown is triggered by local heating effects caused by highly localized processes taking place at macro and micro-defects in the oxide (electrolyte-filled fissures, micropores, flaws) [46,58]. The voltage keeps increasing continually, but the voltage–time slope decreases, and a large number of bright spots (micro-discharges) appear over the whole surface. The slope decreases as a consequence of the relatively low voltage required to maintain the same total current density, because of the dependency of the electron’s current density on the anodic coating thickness. Further, the third stage is reached when the voltage is relatively constant, with a voltage–time slope near zero, since the total current density is practically independent of the anodic layer thickness, as the fraction of electronic current density in the total current density becomes dominant [46,58].

In turn, in potentiostatic mode, when a constant potential is applied during the MAO process carried out in AC mode, the same three stages are observed (Figure 3B) [55]. According to Ohm’s law, for this situation, the first stage is characterized by a sharp decrease in the current density, which corresponds to the moment in which the surface oxidation forms the anodic coating. Then, in the second stage, the current density continually decreases, but with a lower slope, corresponding to the breakdown and beginning of sparking. Finally, the current density is nearly constant.

In another perspective, throughout the MAO process carried out in AC mode using a silicate electrolyte to modify a Ta surface, the cell-potential–time responses (Figure 3C) also display an increase in the positive potential (anodic potential), whilst the anodic layer grows (also called barrier layer) until the first inflection occurs. Therefore, fine sparks are initiated and the breakdown starts [63]. A second inflection occurs and the potential rises slower, indicating that the sparks get more intense. In good agreement, the negative potential (cathodic potential) increases during the growth of the barrier layer, reaching a plateau, followed by a potential escalation coincident with the second inflection in the anodic potential. Thus, the potential increases slowly, likewise to the anodic potential.

In particular, Sowa et al. [23,64] studied the influence of two steps of the MAO process carried out in DC mode on the Ta surface. The MAO process was initiated in galvanostatic mode (the first step) under a constant current density, and then, when the process reached the desired voltage, it was switched to potentiostatic mode (second step) under a constant applied voltage. The recorded voltage–time (during the galvanostatic mode as the first step) and the current-density–time curves (during potentiostatic mode) show a strong influence of the electrolyte composition and concentration on the MAO process. As a first notice, when adding the calcium formate (Ca(HCOO)_2_) or the magnesium acetate (Mg(CH_3_COO)_2_) to calcium hypophosphite (Ca(H_2_PO_2_)_2_), the electrolyte conductivity increases. Indeed, there are more charge carriers in the solution; thus, the resistivity decreases, and, consequently, larger and more intense sparks appear at lower voltages, creating larger pores and increasing the current density [23,64]. In agreement, using a different electrolyte composition, the MAO process is greatly influenced by the electrolyte concentration [62]. In effect, the third region of the V-t curve is better discerned for 0.1 and 0.5 M K_2_SiO_3_ since the most concentrated electrolyte (1 M K_2_SiO_3_) voltage oscillations are verified, which correlates with the necessary time to reach the maximum voltage of 400 V, as it is dependent on the K_2_SiO_3_ concentration (increasing the electrolytes’ concentration, more time is required).

Hence, it is possible to summarize that the stages of the MAO process are dependent on the electrolyte composition and concentration, but are independent of the operation mode (DC or AC).

### 2.2. Gas Evolution Phenomena

**Figure 4:**
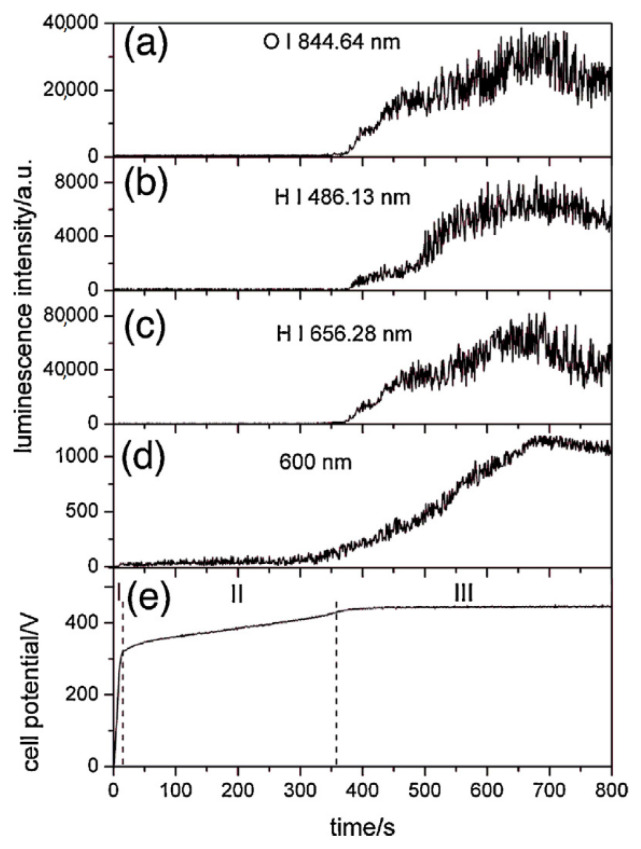
The temporal variation in the luminescence intensity of (**a**) oxygen and (**b**,**c**) hydrogen lines, (**d**) continuum radiation at 600 nm, and (**e**) cell potential. Adapted with permission from Ref. [61]. Copyright 2015 Elsevier.

Stojadinović et al. [61] reported two models that explain the formation of sparks. The first model suggests that the appearance of spark discharge comes as a consequence of the dielectric breakdown of the oxide in the presence of a strong electric field. The second model considers spark discharges as gas discharges occurring in micropores of the oxide, since the formation of a gas phase in micropores is generated by the initial dielectric breakdown in the bottom of the micropores. The authors reported that both models can occur and can be distinguished during the MAO treatment. To achieve that, the MAO process was carried out at 70 mA/cm^2^ using a phosphoric acid (H_3_PO_4_) electrolyte. In the first model, considering the V-t curve (Figure 4e), after the anodic coating growth (first stage), the second stage is verified by the absence of ionic and atomic lines observed in the luminescence spectrum, suggesting that, at the beginning of spark discharge, there is continuum radiation (Figure 4d). In the third stage, when the electric field reaches a critical value and the electronic current is high enough to cause gas discharge occurrence in the micropores, strong ionic and atomic emission lines appear, ascribed to O_2_ (Figure 4a) and H_2_ species (Figure 4b,c), in agreement with the second model [61]. In summary, the first and second stages of the V-t curve are explained by the first model, whereas the third stage is described by the second model.

Concomitant with micro-discharges, during the MAO process, carried out in pulsed bipolar current density regime, the anodic pulses are responsible for multiple electrode processes, such as anodic coating formation and oxygen evolution, whereas the cathodic pulses lead to hydrogen evolution. The gas bubbles contain hydrogen and oxygen, both generated electrochemically and by the dissociation of water molecules [63]. In this sense, some investigations have been carried out to understand the behavior of gas bubbles and spark formation, recording real-time images during the MAO process.

**Figure 5:**
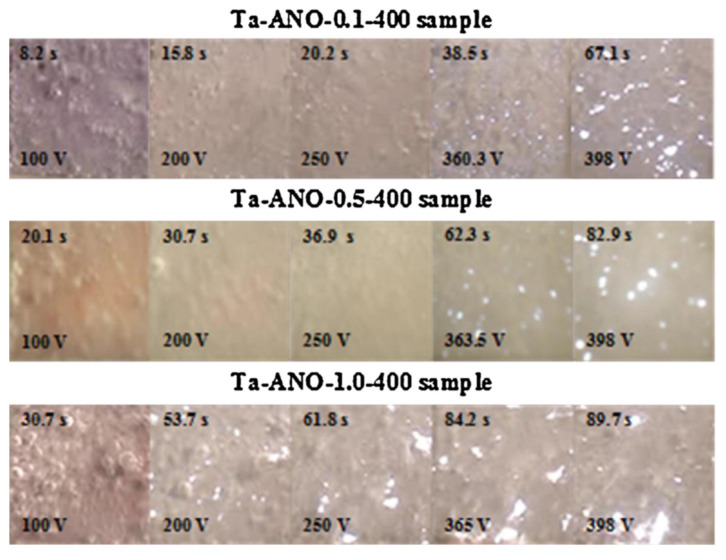
Real-time images of the MAO process of Ta. Reprinted with permission from Ref. [62]. Copyright 2013 Elsevier.

Sowa et al. [62] studied the gas bubbles’ formation and evolution through time, resulting from an MAO process on Ta surfaces. The real-time images (Figure 5) demonstrate that, first, the bubbles are formed, cover the surface, and, while their density decreases, short micro-discharges start to be uniformly distributed over the whole surface, which decreases in number and increases in both diameter and intensity with an increasing time and voltage. For more concentrated electrolytes, the sparks start at lower voltages [62], as mentioned before [23,64].

**Figure 6:**
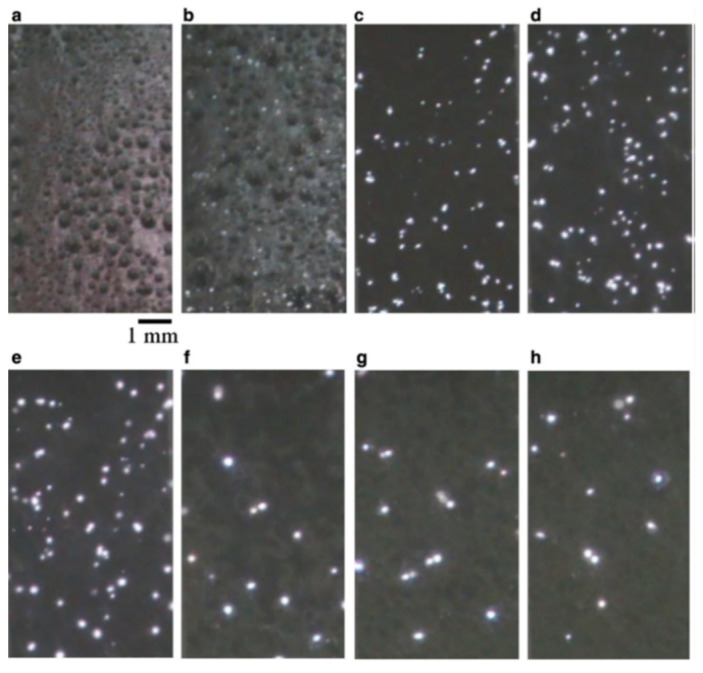
Real-time images of the MAO process of Ta using tungstosilicic acid electrolyte at (**a**) 10 s, (**b**) 20 s, (**c**) 1, (**d**) 3, (**e**) 5, (**f**) 15, (**g**) 30, and (**h**) 45 min. Reprinted with permission from Ref. [58]. Copyright 2011 Elsevier.

On the contrary, Stojadinović et al. [58] carried out the MAO process at 70 mA/cm^2^ using tungstosilicic acid (0.001 M H_4_SiW_12_O), showing different stages of the process with distinct micro-discharge characteristics in the real-time images. Initially, an intensive gas formation is observed immediately after a few seconds of the process (Figure 6a). After 20 s, small micro-discharges already start to be visible, simultaneous with bubble growth evolution (Figure 6b). During the MAO process, as already mentioned, the size of the micro-discharges becomes larger at a reduced number (Figure 6c–f). Additionally, the spatial density of the micro-discharges reaches the maximum after about 2.5 min; then, the spatial density is substantially reduced during the following 5 min, achieving a plateau. In good agreement, the percentage of the oxide area under active discharge sites also has a maximum after 2.5 min, which continually decreases with time. Thus, relatively small discharges occur during the MAO process. The behavior of the micro-discharges is related to the number of micro-discharge sites through which a higher anodic current can pass, since the number of pores decreases whereas their size increases with the MAO duration. The optical emission spectroscopy used to detect the presence of self-absorption for Balmer lines in MAO spectra displays a strong line ascribed to H_α_, whereas the other two weaker Balmer lines are associated with H_β_ and H_γ_. The analysis of the H_β_ line shape indicates the presence of two types of discharges with a relatively low electron number density (Ne) of around 0.9 × 10^15^ cm^−3^ and 2.2 × 10^16^ cm^−3^, respectively, due to the high melting temperature of Ta (compared with Al, which has three types of micro-discharges). This prevents the formation of a metallic plasma in microchannels through the oxide coating.

In a bipolar pulsed current MAO process, the Ta surface presents an initial purple interference color of a thin anodic coating in the first few seconds. This is accompanied by the presence of a gas that consists of electrochemically generated hydrogen produced during the cathodic polarization and possible oxygen created over the anodic polarization [63]. Then, the whole surface has numerous white and fine sparks that reduce in number and increase in intensity, turning to an orange hue. In addition, more energetic gas evolution and a wider dispersion of bubbles into the electrolyte arise in the presence of strong discharges.

### 2.3. Growth Mechanism of Ta_2_O_5_ Anodic Coating

**Figure 7:**
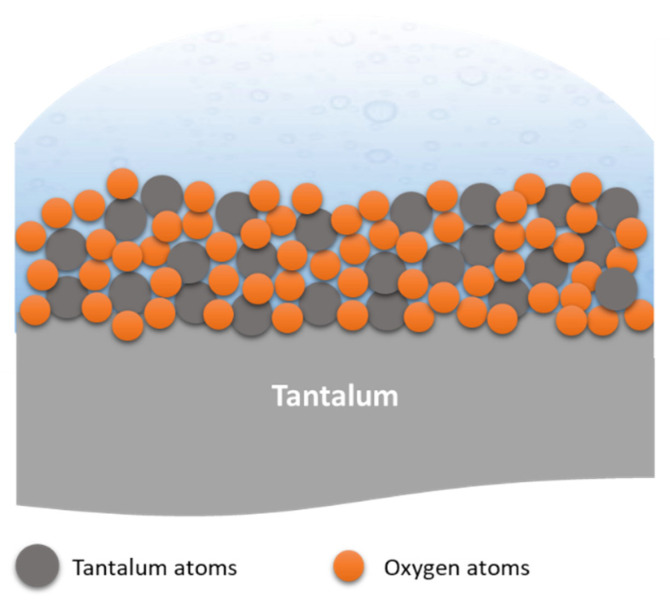
Schematic illustration of Ta_2_O_5_ coating formation resulting from the chemical reaction described in Equation (3).

During the Ta_2_O_5_ anodic coating formation, several interactions occur at different interfaces. For instance, the oxide formation mechanism starts with the oxide coating growing at both Ta/oxide and oxide/electrolyte interfaces. This results from the migration of O^2−^, OH^−^ (down from the electrolyte/oxide interface), and Ta^5+^ ions (up from the metal/oxide interface) across the oxide, assisted by a strong electric field. Consequently, a small amount of the electrolyte is incorporated into the oxide at the oxide/electrolyte interface. The main chemical reaction at the metal/oxide interface is defined by Equation (1), whereas the reaction at the oxide/electrolyte interface is defined by Equation (2), and the overall reaction is defined in Equation (3) [46] (Figure 7).

(1) $$\mathrm{Ta}\to {\mathrm{Ta}}^{5+}+5e^{-}$$

(2) $$4H_{2}O-4e^{-}\to O_{2}↑+4H^{+}+2H_{2}O$$

(3) $$2{\mathrm{Ta}}^{5+}+5H_{2}O\to {\mathrm{Ta}}_{2}O_{5}+10H^{+}$$

**Figure 8:**
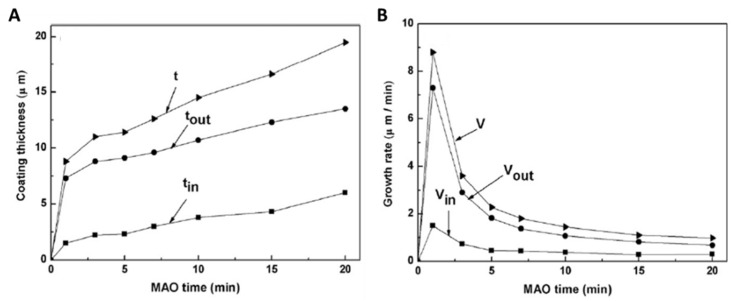
MAO-treated Ta_2_O_5_ coating (**A**) thickness growth (total—t; outward—t_out_, inward—t_in_) and (**B**) growth rate (total—V; outward—V_out_, inward—V_in_) as time progresses. Reprinted with permission from Ref. [65]. Copyright 2013 Elsevier.

According to the measurements of the total thickness (t), outward growth thickness (t_out_), and inward growth thickness (t_in_) (Figure 8A) on the cross-sectional SEM micrographs, during the MAO process, the total growth rate (V) variation in the anodic thickness (Figure 8B) indicates that the growth process of MAO coatings can be divided into three distinct periods [65]. In the first minute of the MAO process carried out at a bipolar pulsed voltage, a huge increase in the total thickness occurs, as well as in the total growth rate V of the anodic coating, so the outward growth rate (V_out_) is much higher than the inward growth rate (V_in_). Thus, the outward growth rate dominates the anodic growth in the first stage. In the second stage, after 1 min of MAO, the total thickness increases more slowly and the total growth rate declines abruptly.

The descendent tendency is more pronounced in the outward growth rate than in the inward growth rate. Consequently, the outward growth behavior slows down, whereas the inward growth is kept constant. In the final stage, the total thickness continues increasing slightly and the total growth rate drops more gradually. The outward growth rate continues to decrease gradually and the inward growth rate continuously increases, as the downward tendency of the outward growth rate is still more pronounced. In fact, in this period, the increment of the outward growth thickness is lower than the increment of the inward growth thickness, indicating that the inward growth has a greater contribution to the total thickness in the final stage, which is contrary to the first stage. The described growth behavior was observed for the MAO treatment carried out at 350 and 450 V.

In more detail, during the MAO process, sparks or plasma micro-discharges in an aqueous solution are responsible for an ionized gaseous media formation from the solution over the metallic surface through the plasma chemical interactions [65]. Thus, discharge and heat-affected zones are generated, and both play a fundamental role in adsorption centers, supplying conditions to the solute anions being adsorbed on the anodic oxide coating. Hence, under a strong electric field, the anions diffuse from the electrolyte to the metal/oxide interface, reaching the metal cations to form a ceramic coating, whereas the cations diffuse from the metallic surface to the oxide/electrolyte interface and react with anions to form the anodic layer. Using an electrolyte mixture of calcium acetate and β-glycerophosphate [65], it is reported that, in the first stage of the MAO process, there are numerous fine sparks over the whole surface, providing several prone adsorptive zones for anions (such as O^2−^ and PO_4_^3−^) to enter the oxide coating, and plasma-chemical reactions for the cations (such as Ta^5+^ and Ca^2+^) to travel across the oxide and compound with the anions into the outer layer; thus, the outward growth dominates this phase. Along the MAO time, the number of sparks decreases, and their size increases, while the diffusion and electron migration are blocked by the increase in the anodic coating thickness. The inward migration of anions from the electrolyte to the metal/oxide interface is easier than Ta^5+^ from the metallic substrate to the oxide/electrolyte interface. Thus, inward growth gradually becomes dominant in growth behavior at the latter stages.

**Figure 9:**
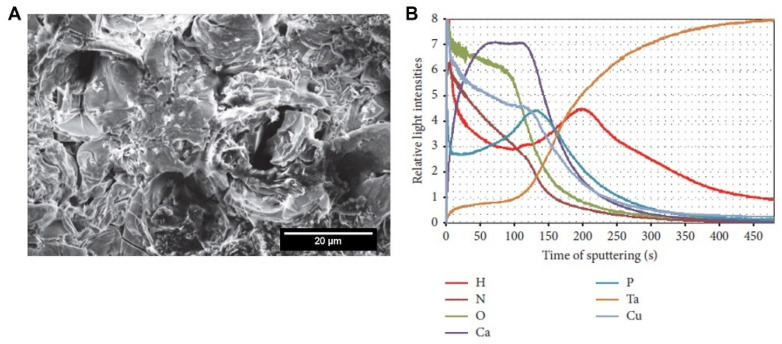
(**A**) Surface morphology of MAO-treated Ta surface. (**B**) Relative light intensities of tantalum, phosphorus, calcium, copper, oxygen, and nitrogen anodic coating formed on tantalum obtained by the GDOES method. Adapted from Ref. [66].

To better understand the ionic incorporation into the anodic coating, Rokosz et al. [66] studied the Ta surface modification by MAO using an electrolyte mixture of copper nitrate (Cu(NO_3_)_2_) and calcium nitrate (Ca(NO_3_)_2_) in phosphoric acid (H_3_PO_4_). The morphology achieved is neither porous nor flat (Figure 9A), and its top surface is mainly composed of calcium phosphates, such as CaHPO_4_ and/or Ca_3_(PO_4_)_2_, and copper phosphates. Through the GDOES profile (Figure 9B), concerning the erosion time (not directly related to the depth penetration, as it is difficult to accurately estimate it on rough and porous surfaces), the authors propose a new model of the MAO-treated Ta surface composed of three sublayers. The top one (0–40 s) is enriched by P, copper (Cu), oxygen (O), nitrogen (N), and hydrogen (H), with a small content of Ta and Ca. The second one, which corresponds to the erosion time between 40 to 80 s, contains Ca, Cu, and O. The third sublayer is divided into four different zones. The first, from 80 to 125 s, is enriched with Ca, Cu, and O, with depletion of Ta and H, and an increasing trend of P and decreasing trend of N are recorded. At 125 to 150 s (second zone), the P content achieves a maximum with an increase in Ta and H, whereas Ca, Cu, O, and N tend to decrease. Then, between 150 and 270 s, a maximum signal of H is detected, whereas Ta continues increasing, and the others continue decreasing. After 270 s, all signals decrease, except Ta. In summary, the Ca and Cu are distributed practically over the whole volume of the anodic coating, whereas O, N, and H are mainly at the oxide surface and Ta is mainly on the oxide bottom.

## 3. Ta Surface Functionalization by MAO

MAO is a process well established and studied on Ti surfaces; however, there are just a few published papers on Ta surfaces. Table 1 displays papers on MAO-treated Ta surfaces and is divided into the two morphologies that can be obtained: porous and non-porous structures. As mentioned before, the MAO process may induce non-uniform and non-homogenous porous structures by one side, but it can also form non-porous surfaces, such as a compact oxide coating with some pores and/or discharge channels [67], cracks, and “alveolar” and “pancakes” features.

Although the studies on Ta surfaces modified by MAO are scarce, most of them are based on the formation of porous structures, which allows for the incorporation of Ca and P species from the electrolyte, developing functional surfaces for biomedical applications as shown in Table 1. Generally, in order to perform MAO on Ta-based surfaces, most of the papers report an electrolyte that is composed of a mixture of calcium acetate (CaA) and β-disodium glycerophosphate (β-GP) to promote Ca and P incorporation into the anodic coating, respectively.

In a recent review paper about Ta modification by MAO, the authors try to relate electrolyte composition and applied voltage with the morphology achieved and the possible growth mechanism [68], yet the assumptions are not conclusive.

**Table 1:** Review table of the different types of Ta nanostructures after MAO treatment under different conditions.

Type of Microstructures	Electrolyte	Working Conditions	Time (s)	Temperature (°C)	Pre-Treatment	Applications	References
**Porous**	0.2 M CaA + 0.02 M β-GP	AC: V+ = 350, 400, 450, 480 V, V− = 70 V, 100 Hz, duty ratio of 26%	600	20	Polished with abrasive papers; ultrasonically cleaned in acetone, ethanol, and DI water	Biomedical	[69]
		AC: V+ = 350, 450 V, V− = 70 V, 100 Hz, duty ratio of 26%	60–1200	20	Polished with abrasive papers; ultrasonically cleaned in acetone, ethanol, and DI water	Orthopedic implants	[65]
		AC: V+ = 470 V, V− = 100 V, 100 Hz, duty ratio of 26%	300			Biomedical	[70]
		AC: V = 250–480 V, 100 Hz, duty ratio of 40%	300, 600, 900 and 1800	30	Polished with abrasive papers; ultrasonically cleaned in acetone, ethanol, and DI water; dried at 60 °C	Orthopedic implants	[71]
		AC: 160–300 V	60–300	RT	Washed in DI water; ultrasonic clean in acetone; cleaned in ethanol and air-jet dried	Dental implants	[72]
		AC: 350, 450 and 500 V, 100 Hz, duty cycle 60%	60–600	25–60	Sonicated in acetone, isopropyl alcohol, and DI water baths	Biomedical	[55]
	A: 0.35–0.7 M CaA B: 0.7 M CaA + 0.04–0.08 M β-GP C: 0.7 M CaA + 0.08 M β-GP + 0.01–0.1 M MgA	DC: 150–200 V	1800	RT	Ultrasonically cleaned in benzine and ethanol for 5 min each; rinsed in DI water and dried in air	Dental implants	[73]
	0.35 M CaA + 0.12 M β-GP	DC: 200 V	1800	RT	Ultrasonically cleaned in ethanol and DI water for 5 min each	Dental implants	[74]
	(0.1, 0.5, 1.0 M) K_2_SiO_3_ + 5 g/dm^3^ KOH	DC: 0.1 A/dm^2^ up to 100, 200 or 400 V	120		Etched in 1 M HF and 4 M H2SO4; cleaned in DI water and ultrasonically cleaned in propanol and DI water	Orthopedic implants	[62]
	A: 0.5 M Ca(H_2_PO_2_)_2_ 0.5 M Ca(H_2_PO_2_)_2_ + 1.15 M Ca(HCOO)_2_ C: 0.5 M Ca(H_2_PO_2_)_2_ + 1.15 M Mg(CH_3_COO)_2_ D:0.5 M Ca(H_2_PO_2_)_2_+ 1.5 M Mg(CH_3_COO)_2_	DC: 150 mA/cm^2^ up to 200, 300, 400 or 500 V	300		Polished with abrasive paper; etched in 4 M H_2_SO_4_ and 1M HF; ultrasonically cleaned in DI water	Biomedical	[23]
	A: 0.5 M Ca(H_2_PO_2_)_2_ B: 0.5 M Ca(H_2_PO_2_)_2_ + 1.15 M Ca(HCOO)_2_ C: 0.5 M Ca(H_2_PO_2_)_2_ + 1.15 M Mg(CH_3_COO)_2_	DC: 150 mA/cm^2^ up to 200, 300 or 400 V					[64]
	0.001 M H_4_SiW_12_O_4_	DC: 70 mA/cm^2^	15–2400	21	Ultrasonically cleaned in acetone, ethanol, and DI water and dried in a warm air stream	Catalysis and semiconductor	[46]
						The automotive industry, aerospace industry, and gas and oil industries	[58]
	30 mL HF (4%) + 5 mL NH_4_F + 1 g glycerin + 3g EG + 2 M H_3_PO_4_	DC: 250 V	900		Polished with abrasive papers and in Al_2_O_3_ suspension	Biomedical	[75]
	12 g/L Na_2_SiO_3_ +10 g/L NaOH + small amount of additives (EDTA)	AC: 450 V, 1000 Hz, duty cycle 20%	300	20	Polished with abrasive papers to a mirror finish; ultrasonically cleaned in acetone, ethanol, and DI water for 5 min; and dried	Orthopedic implants	[76]
	0.3 M Ca(CH_3_COO)_2_.H_2_O + 0.1 M Na_3_PO_4_	AC: 1.5 A, 500 Hz, duty cycle 10%	480	26 ± 3	Ultrasonically cleaned in acetone, ethanol, and DI water	Biomedical	[77]
**Non-Porous**	A: 1 g/L Na_2_SiO_3_.5H_2_O +1 g/L KOH B: 5 g/L Na_3_PO_4_ + 1g/L KOH	AC: 0.085 A/cm^2^	1200	30		Biomedical	[67]
	300g Ca(NO_3_)_2_ + 300 g Cu(NO_3_) in 1 L of H_3_PO_4_ (85%)	DC: 450 V	180	20	Polished with abrasive papers	Biomedical and catalysis	[66]
	10 g/L Na_2_SiO_3_.9H_2_O + 1 g/L KOH	AC: j+ = 0.22 A/cm^2^, j− = 0.11 A/cm^2^, 1000 Hz, duty cycle 20%	1500		Polished with abrasive papers; degreased in ethanol and rinsed in DI water; dried in warm air	Biomedical	[63]
	A: 0.1 M H_3_PO_4_ B: 0.1 M oxalic acid	DC: 30–70 mA/cm^2^	10–600	21–30	Ultrasonically cleaned in acetone	Capacitor dielectric, gate insulators in MOS devices	[61]

## 4. Ta_2_O_5_ Surfaces for Biomedical Applications

### 4.1. Properties of MAO-Treated Ta Surfaces

#### 4.1.1. Surface and Cross-Sectional Morphology

**Figure 10:**
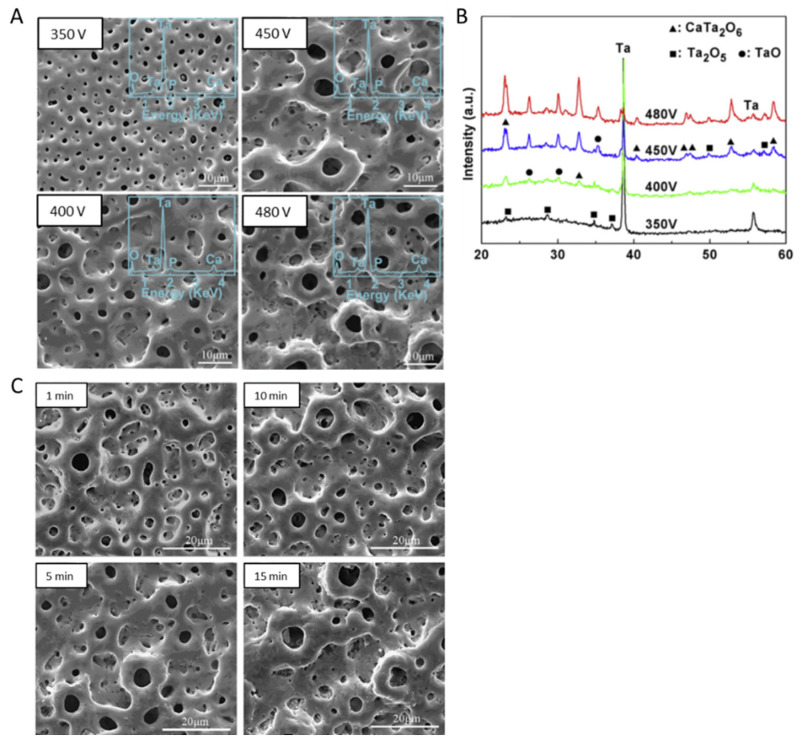
(**A**) Surface morphologies of Ta_2_O_5_ treated by MAO at different applied potentials with EDS spectra inset (scale bar 10 μm), and (**B**) the respective XRD patterns. Adapted from [69], Copyright 2016 Elsevier. (**C**) Surface morphologies of Ta_2_O_5_ treated using MAO process at 450 V for different process durations, scale bar 20 μm. Adapted with permission from Ref. [65]. Copyright 2013 Elsevier.

**Figure 11:**
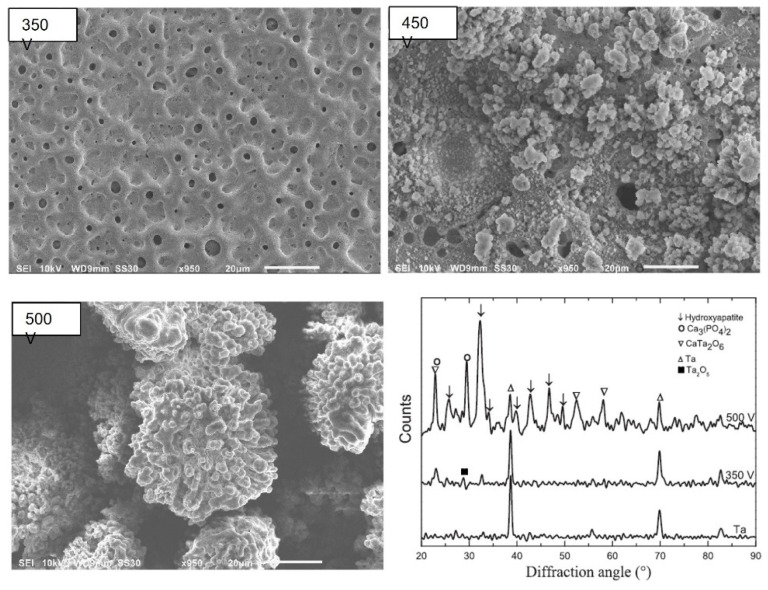
Surface morphologies of Ta_2_O_5_ treated by MAO at different applied potentials, scale bar 20 μm, and XRD patterns for untreated Ta and MAO-treated Ta at 350 and 500 V. Adapted with permission from Ref. [55]. Copyright 2019 Elsevier.

As mentioned above, the most reported electrolyte composition is a mixture of 0.2 M CaA and 0.02 M β-GP as precursors for Ca and P incorporation, respectively (Table 1). This electrolyte leads to a micro-porous surface, with diameters ranging from 1 to 5 μm [55], where the pores are distributed over the surface [55,70,71] and its distribution is related to the preferential growth and the dielectric breakdown of the oxide coating with the evolution of oxygen and aqueous vapor [65]. The number of pores decreases whereas the size of the pores increases with the applied voltage (Figure 10A) [69] and with MAO time (Figure 10C) [65] as a consequence of the proximity of the small discharge channels connection [71]. On the other hand, the growth of clusters with fractal-like columnar structures (dendritic structures) on top of the micro-porous surfaces is observed when increasing the applied voltage up to 450 V, and, at higher voltage (500 V), the dendritic becomes larger than 20 μm, covering the surface porosity (Figure 11) [55]. In these studies, the MAO treatment is carried out at 450 V during 600 s, but the process differs in the duty cycle and the surface pre-treatment (i.e., polishing) (Table 1): using a polished Ta surface and a duty cycle of 26%, only porous Ta_2_O_5_ surfaces are achieved (Figure 10A,C) [65,69], whereas, using an unpolished Ta surface and a duty cycle of 60%, dendritic structures appear (Figure 11) [55].

We have recently reported the Ta surface treatment by MAO at 200 V, and that using an identical electrolyte composition but with a higher concentration develops an oxide layer with a porous morphology similar to the morphology obtained by Wang et al. [65,69], without dendritic structures [73,74]. Moreover, the influence of the electrolyte composition (CaA, CaA + β-GP, CaA + β-GP + MgA) and concentration on the morphology was also studied. The CaA concentration increase leads to an increase in porosity. Although the morphology variation induced by the β-GP concentration increase is not as significant as the one induced by CaA concentration, for the same CaA concentration, when β-GP is added to the electrolyte, increasing the process reactivity increases the porosity [73].

In good agreement with the morphological results of MAO-treated surfaces using an electrolyte composed of a mixture of CaA and β-GP, when the electrolyte used is composed of Ca(H_2_PO_2_)_2_ and Ca(HCOO)_2_ or Mg(CH_3_COO)_2_, the diameter of the pores directly increases with an increase in the applied voltage [23]. Using more conductive electrolytes, larger pores are created, and, up to 500 V, some dendritic structures are formed, which are correlated to the more energetic sparks that generated these morphologies due to the partial evaporation of the electrolyte combined with thermal and plasma effects. The anodic coating displays a multilevel porosity since it shows small pores in its internal layer, and, together with the thickness, they are revealed to be voltage and electrolyte-dependent since the Mg-enriched electrolyte is responsible for the thickness’s decrease as a consequence of the electrolyte concentration increase [23]. This may be related to the lower standard potential of Ca concerning Mg or due to counter ions used in their respective salts [64].

The anodic coating generally shows a uniform thickness and with no discontinuity at the oxide coating/substrate interface [65,70], with a compact inner region and a porous outer region [55,77]. Yang et al. [77] demonstrated that the dense inner layer is about one-third of the total oxide thickness when the MAO process uses an electrolyte composed of calcium acetate monohydrate and sodium phosphate. This inner layer has no pores or discontinuities, indicating a strong bonding between the ceramic coating and the Ta substrate. The authors also show that the current density has an important effect on the oxide thickness. They study the MAO treatment of two Ta surfaces with different total surface areas: the surface with the lowest total surface area results in the highest current density and, consequently, the thickest oxide coating is formed with more small pores (≤1 μm) as a consequence of more severe micro-arc reactions [77].

**Figure 12:**
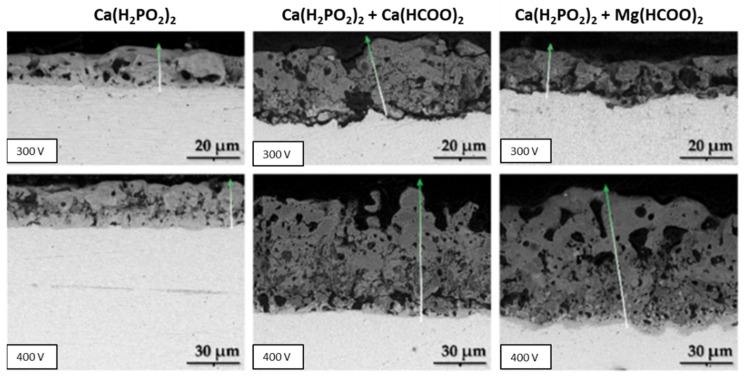
SEM cross-sectional images of the MAO-treated Ta surfaces obtained from different electrolyte compositions and at different limiting voltages. Adapted from Ref. [64].

Using an electrolyte mixture of Ca(H_2_PO_2_)_2_ and Ca(HCOO)_2_ or Mg(CH_3_COO)_2_, it was found that the anodic coating microstructure is also revealed to be dependent on the applied voltage [64]. In this case, the microstructure can be divided into two or three sublayers: the outer porous layer; the inner compact barrier layer at the substrate/oxide interface; and, for MAO at voltages higher than 400 V, a third highly porous intermediate sublayer appears, and is more porous than the outer layer (Figure 12). This microstructure can be formed due to the solidification and oxidation of the molten substrate during the MAO process.

Additionally, the presence of minor cracks on the porous surfaces was reported by Zhao et al. [71], which can be related to the thermal stress caused by the high temperature generated by the discharges during the MAO process.

**Figure 13:**
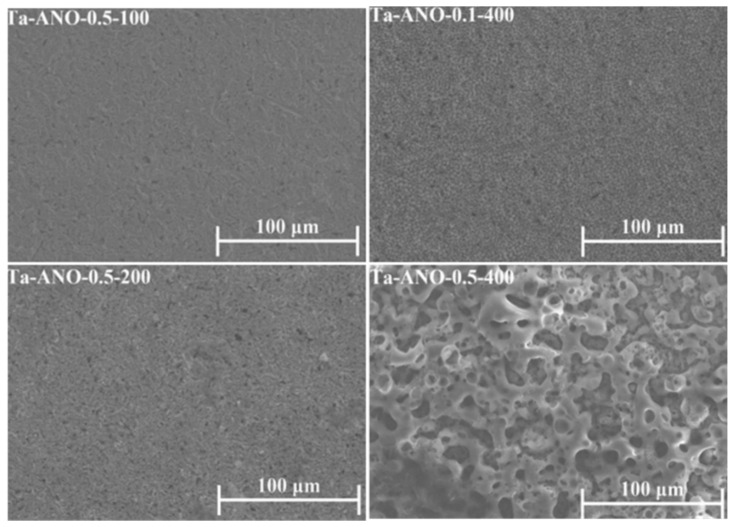
Surface morphologies of Ta_2_O_5_ prepared by MAO under different conditions: 0.5 M K_2_SiO_3_ at a range of 100 to 400 V, and 0.1 M K_2_SiO_3_ at 400 V. Adapted with permission from [62]. Copyright 2013 Elsevier.

Another key point influencing the MAO Ta-treated surfaces is the electrolyte composition. Other than the Ca-enriched electrolytes, silicate and phosphate solutions are also commonly used in MAO treatments (Table 1). MAO-treated Ta surfaces, using an electrolyte mixture of different concentrations of potassium silicate (K_2_SiO_3_) and potassium hydroxide (KOH), show a porous structure when the applied voltage is above the dielectric potential [62]. The thickness of the oxide coating and the diameter of the pores are dependent on the electrolyte concentration and applied voltage, so much so that the increase in K_2_SiO_3_ concentration or the applied voltage produces thicker surfaces and larger pores (Figure 13). Thereafter, in a thicker coating, as expected, higher energy is needed for the current to pass through. In this situation, the current is localized at weak points of the oxide coating and, consequently, the diameter of the channels increases. The anodic coating shows a typical structure with two sublayer regions: a thin and compact inner layer adjacent to the Ta substrate, and a porous outer layer. Similar morphology was obtained with an electrolyte composed of sodium silicate (Na_2_SiO_3_) and sodium hydroxide (NaOH) [76].

**Figure 14:**
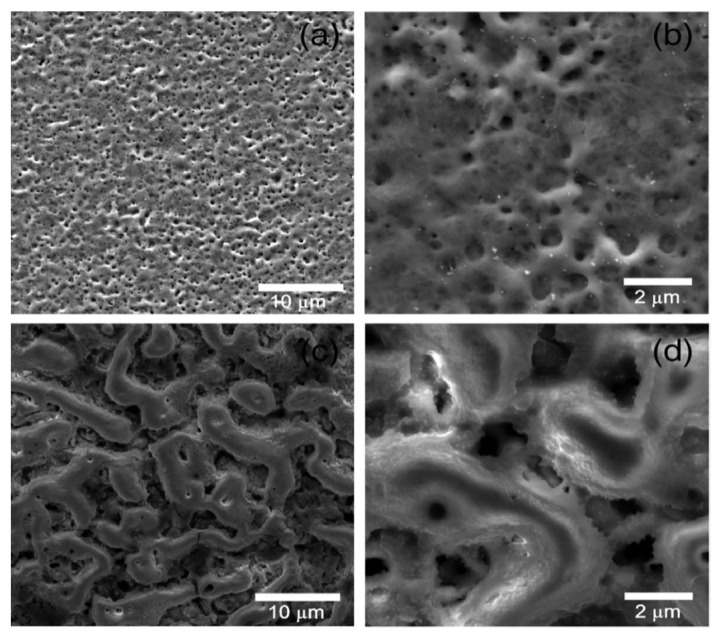
Surface morphology of (**a**,**b**) microcrystalline Ta and (**c**,**d**) nanocrystalline Ta after MAO treatment. Reproduced with permission from Ref. [75]. Copyright 2020 John Wiley and Sons.

Both MAO-treated nanocrystalline and microcrystalline Ta surfaces using an electrolyte composed of hydrofluoric acid (HF), ammonium fluoride (NH4F), glycerin, ethylene glycol (EG), and H_3_PO_4_ at 250 V result in the formation of porous oxide coatings with good adhesion to the substrate [75]. For microcrystalline Ta, the oxide coating morphology is characterized by the formation of small cylindrical pores (Figure 14a,b), whereas, for the nanocrystalline Ta, a thicker oxide with irregular channels is obtained (Figure 14c,d). These results reveal that the achieved morphology depends on the substrate crystalline structure: the microcrystalline Ta is only composed of the Ta phase, whereas additional phases appear (Ta, TaC, TaB_2_, TaO_2_) resulting from the nanocrystalline Ta bulk preparation.

In summary, the coating thickness and its porosity increase with the applied voltage and with the electrolyte concentration (which is responsible for exerting less resistance to the current flow, intensifying the process [64]), as described in the MAO-treated Ta surfaces under different electrolyte composition and operation modes (AC or DC) [23,55,64,69], as well as increasing with the MAO duration [55,65]. The thickness and porous morphology of the resulting Ta_2_O_5_ coating also depend on the total surface area [77] and crystalline structure of the Ta substrate [75].

As mentioned before, besides these Ta_2_O_5_ porous morphologies achieved after MAO treatment, other non-porous morphologies can also be obtained.

**Figure 15:**
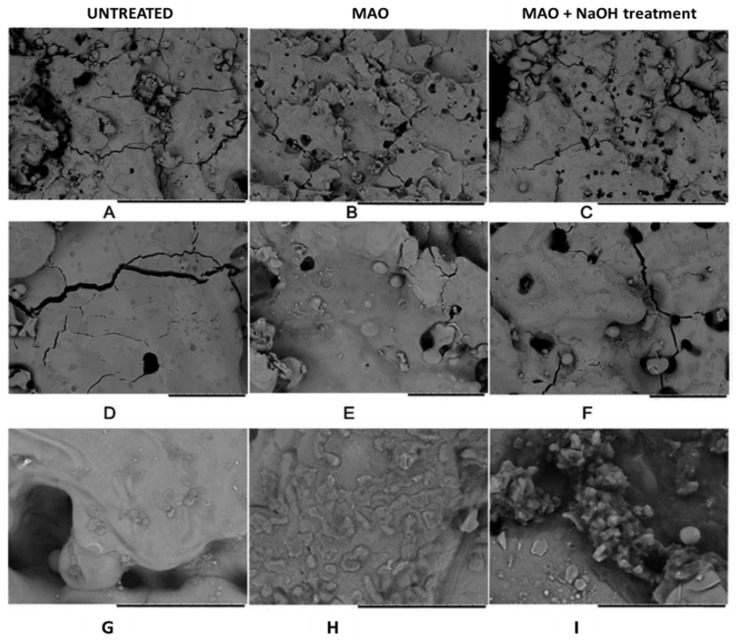
Surface morphologies of (**A**,**D**) untreated Ta surface, (**B**,**E**) Ta_2_O_5_ prepared by MAO using the phosphate solution, and (**C**,**F**) with NaOH treatment.; (**G**–**I**) after SBF soaking. Scale bars (**A**–**C**) 200 μm, (**D**–**F**) 30 μm, and (**G**–**I**) 20 μm. Adapted with permission from Ref. [67]. Copyright 2014 Royal Society of Chemistry.

MAO-treated Ta surfaces (on a polyurethane foam) using an electrolyte mixture of sodium metasilicate (Na_2_SiO_3_.5H_2_O) or trisodium phosphate (Na_3_PO_4_) and KOH are submitted to an alkaline post-treatment to evaluate the effect on the surface bioactivity [67]. The authors reported that the pores (or holes) are already observed on the untreated Ta surface (Figure 15A,B). After MAO treatment, the surface shows relatively more holes, as expected (Figure 15B,E), which are completely different (small and spaced holes on a cracked surface) from those previously reported. Therefore, this MAO-treated Ta surface is, here, considered as a non-porous surface. Additionally, many cracks in the boundary grains are observed before and after the MAO treatment. With the alkaline (NaOH) post-treatment, where the samples are soaked in NaOH at 60 °C for 24 h, more holes are observed (Figure 15C,F) when compared with both untreated Ta and MAO-treated Ta surfaces, and cracks are still detected.

**Figure 16:**
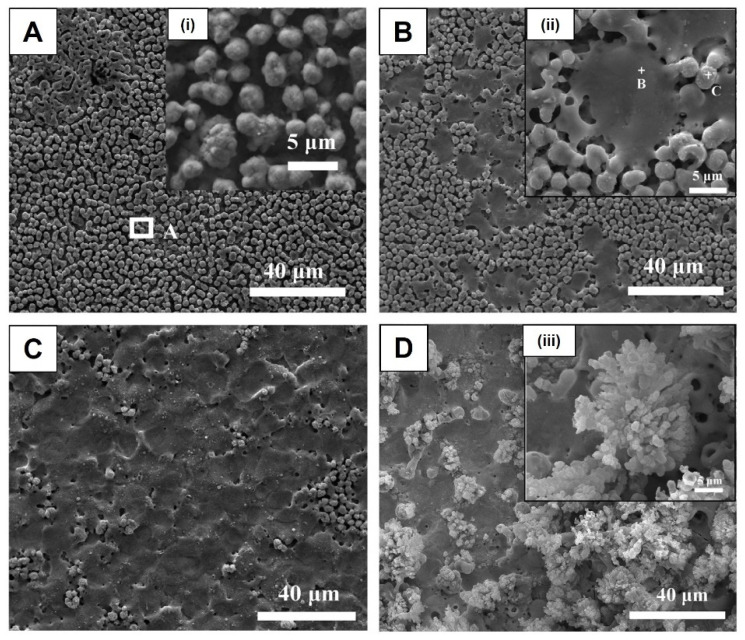
Surface morphologies of MAO-treated Ta surface using a silicate electrolyte in a bipolar pulsed current for (**A**) 120 s, (**B**) 140 s, (**C**) 180 s, and (**D**) 600 s (scale bar: 40 μm). Inset high magnification SEM micrographs of the (**i**) **A**, (**ii**) **B** and (**iii**) **D** morphologies (scale bar: 5 μm). Adapted with permission from Ref. [63]. Copyright 2020 Elsevier.

The Ta surface modified by MAO under a pulsed bipolar current using a silicate electrolyte (more concentrated than the previously reported silicate solution) shows transitional morphologies depending on the treatment time [63]. An initial nodular morphology is observed (Figure 16A), and then smooth pancakes are formed around the nodules (Figure 16B). These pancakes result from the unquenched discharge channels and, thus, they become dominant with a few nodules over the surface (Figure 16C). These morphological features are identical to those previously reported by Stojadinović et al. [61] in the DC MAO treatment using the H_3_PO_4_ electrolyte. Increasing the MAO time, several coral reef features (with dendritic morphology and nodular branches) arise, and the pancakes are still evident between the coral reef, which is non-uniformly distributed over the surface (Figure 16D) [63]. The presence of the coral reef increases the thickness of the anodic oxide coating.

These morphological transitions are coincident with the transient in cell potential. Before the escalation of the negative potential (Figure 3C: in between the first and second inflection points), the surface is characterized by nodule features and fine sparks, and hydrogen evolution over the whole surface (Figure 16A). Then, the first transition occurs (Figure 3C: after the second inflection point), leading to an escalation in the overpotential for hydrogen evolution and an increase in sparking intensity (change in spark color and gas generation), which creates the pancakes due to material solidification from the quenching discharges, such as nodular material (Figure 16B,C). The sparking intensification results from a gradual thickening of the pancake covering the surface defects, resulting in a current concentrating in fewer favored regions for dielectric breakdown. The second transition, associated with a further increase in the overpotential, arises more slowly and coincides with the formation of coral reef features (Figure 16D) followed by an increase in the anodic potential associated with the growth of the barrier layer. The growth of this barrier layer involves the anodic and thermal oxidation of the Ta substrate (and molten ejection of Ta_2_O_5_) caused by the stronger discharges and high temperatures.

Thus, an MAO process using silicate and phosphate solutions leads to the formation of a relativity compact oxide coating, and, when increasing the current density, electrolyte concentration, and/or the treatment duration, a nodular morphology with flat pancakes is formed, and, after 600 s, a coral reef morphology can arise.

#### 4.1.2. Surface Chemical Composition

For certain applications, it is desired to dope the anodic coating with specific elements to tailor specific properties. In this regard, the incorporation of Ca and P elements onto the oxide coating has been demonstrated, which increases with the increase in the MAO time and the applied potential (Figure 10A in insets) [55,65,69,71,72,73], as well as when increasing the electrolyte concentration [73,74]. Interestingly, when increasing the β-GP concentration in the electrolyte, a boost in Ca incorporation is also noted, besides the P incorporation increase. This anodic coating doping is related to the phenomena of spark generation and the local increase in temperature, which directly depends on the electrolyte composition/concentration. Moreover, Ca and P incorporation is more effective, as revealed by a higher surface concentration of these elements [73]. In addition, P is only observed in the bulk when the MAO treatment voltage is higher than 450 V, as well as for times higher than 180 s. When the applied potential is increased, electrophoresis becomes dominant and contributes to the increase in the P species’ density near the substrate. This increase, combined with the large available energy provided by the micro-arcs, increases the rate of complex reactions taking place during the MAO process, leading to the formation of calcium phosphates and hydroxyapatite (HAp) [55]. Indeed, the electrolyte ions enter into the anodic coating via diffusion and electrophoresis, in which, Ca^2+^ ions mainly arrive through diffusion, while P (in the form of PO_4_^3−^) moves by electrophoresis along the discharging channels [71]. Additionally, the presence of sodium (Na) and magnesium (Mg) is reported by Goularte et al. [72] on an MAO-treated surface using an electrolyte composed of 0.2 M CaA and 0.02 M β-GP. The authors attribute this fact to the salt deposition during the MAO treatment. Although not explained by the authors, these contaminations can be explained by the precursors’ chemical composition impurities; indeed, β-GP is a common carrier of Na [78]. However, Mg contamination does not have any evident source. In addition, the authors only report the presence of P on the surface, modified mainly at a higher voltage (up to 300 V) and for 5 min of treatment (the highest time) [72]. Still, P absence can be related to both Ta Mα and P Kα peaks overlapping, compromising P identification (which can be qualitatively compared to the untreated Ta surface).

However, as expected, for all solution mixtures of Ca(H_2_PO_2_)_2_ and Ca(HCOO)_2_ or Mg(CH_3_COO)_2_, the relative amount of elemental species’ incorporation increases with a decrease in the Ta content. The P content remains practically static whereas the Ca and Mg contents increase with the applied potential. Nevertheless, the addition of Mg decreases the incorporation content of Ca [23], which can be explained by the competitive incorporation of the cations [78]. Additionally, the results show that the incorporation of the electrolyte species is higher in the outer region of the anodic coating [64].

For the MAO process using a Ca- and P-free electrolyte composed of a combination of K_2_SiO_3_ and KOH, the amount of Si incorporated into the anodic coating increases with the K_2_SiO_3_ concentration [62]. In addition, the K concentration increases towards the oxide coating. The XRD patterns display peaks that are associated with the metallic Ta and Ta_2_O_5_, and, using XPS, the presence of silica/silicate incorporation is confirmed, silica being the dominant component. Using a more concentrated silicate electrolyte with KOH in a bipolar pulsed current, the different morphologies achieved along the MAO process show different chemical compositions [63]. The nodules are constituted by Ta, O, and Si, whereas the pancakes are only composed of Ta and O, and the coral reefs are silicon-rich. Using a silicate electrolyte mixture with NaOH in a bipolar pulsed voltage, the Si is uniformly distributed over the porous Ta_2_O_5_ coating [76].

To sum up, surface modification by the MAO allows for the incorporation of chemical elements present in the electrolyte onto the anodic coating, and this incorporation can be tailored mainly by the electrolyte concentration/composition, applied potential, and process duration.

#### 4.1.3. Roughness

Based on surface functionalities, surface roughness is one of the most important surface properties. For example, roughness at the micro and nano-scale is related to the improvement in biological responses [52].

The MAO process using electrolytes composed of Ca and P induces the formation of porosity, leading to an increase in surface roughness [73,74]. In addition, by increasing the applied voltage, the porosity is enhanced and, consequently, the surfaces become rougher [23,55], mainly when the formation of dendritic structures occurs (Ra > 5 μm) [23]. When adding MgA to the electrolyte, for an applied potential of 200 V, any amendment of the surface morphology and roughness is noticed [23,73].

Similarly, using silicate electrolytes, the surface roughness is revealed to be dependent on the electrolyte concentration and applied voltage, so much so that an increase in K_2_SiO_3_ concentration or applied voltage produces larger pores and consequently rougher surfaces [62]. With a more complex electrolyte, the nanocrystalline Ta surface becomes significantly rougher than the microcrystalline Ta surface using the same anodizing parameters [75], due to the different porous morphologies achieved (irregular channels and small cylindrical pores, respectively).

Any paper on the non-porous MAO-treated Ta surfaces presents a topographical characterization of the obtained surfaces.

To sum up, the MAO process improves the surface roughness as a consequence of larger pores and channel formation, which depends on the electrolyte concentration and applied potential, as well as the substrate crystalline structure.

#### 4.1.4. Surface Wettability

In another area of knowledge, surface wettability is another crucial property, since the hydrophobic/hydrophilic character is a determinant that modulates the surface functionality.

Generally, the surface wettability increases with the MAO treatment using electrolytes enriched with CaA and β-GP [73,74]. On the contrary, Sowa et al. [23] reported a general increase in the water contact angle with the MAO process, although the surface wettability did not show a linear tendency. The authors explain this behavior based on the applied voltage. For lower applied voltages (up to 300 V), the authors assume that some surface features can be ascribed by the Cassie–Baxter state. In this specific case, the pores are too small, not allowing the water droplet to easily penetrate them, forming air pockets. The surface, therefore, presents a hydrophobic behavior. For higher applied voltages (≥400 V), the pores are large enough for easier water penetration, behaving like a Wenzel surface state exhibiting a hydrophilic character. The authors attribute the hydrophobicity increase to morphological changes in the surfaces, and not to chemical modifications. On the other hand, when adding MgA in the electrolyte, the water contact angle increases [23,73], indicating the importance of surface chemistry in the regulation of wettability. The presence of Ca^2+^ and PO_4_^3−^ groups is associated with hydrophilic components, thus enhancing the surface wettability [73]. Similarly, using silicate electrolytes, the MAO-treated Ta surface wettability is also revealed to be dependent on the applied voltage, as increasing the voltage strongly decreases the contact angle, and the surface becomes hydrophilic [62]. The different obtained morphologies by MAO under the same anodizing parameters, as a consequence of Ta substrate crystalline structure, are relevant to the surface wettability [75]. The MAO-treated nanocrystalline Ta surface is super-hydrophilic, whereas the MAO-treated microcrystalline Ta is within the hydrophobicity limit, which can be explained by the different porosity degrees (larger pores and channels, and smaller pores, respectively) (Figure 14), in agreement with the results reported by Sowa et al. [23].

The water contact angle also decreases after the MAO treatment, inducing a non-porous morphology [67]. Although the authors do not explain this phenomenon, as the morphological changes are not significant between the untreated and the MAO-treated Ta surfaces (Figure 15), the modification of the surface chemical composition can influence the surface wettability. The other non-porous morphologies (nodular, pancakes, and coral-reef) are not characterized regarding their wettability.

The results support the idea that both topographic and chemical properties of the MAO-treated Ta surface influence the surface wettability.

#### 4.1.5. Structural Analysis and Phase Composition

The phase composition is another key material property that is influenced by the MAO process [79].

On the diffractograms of Ta surfaces treated with CaA and β-GP, the expected crystallized orthorhombic Ta_2_O_5_ and both bcc and tetragonal Ta (from the substrate) are observed [73,74]. When the applied voltage is higher than 350 V, Ta_2_O_5_ and TaO are detected, as well as another predominant phase related to the presence of calcium tantalate (CaTa_2_O_6_) [55,65,69,70,71] (Figure 10B and Figure 11). Similarly, the oxide surface achieved by the MAO treatment using an electrolyte mixture of CaA monohydrate and sodium phosphate in AC mode is mainly composed of CaTa_2_O_6_, Ta_2_O_5_, and Ta phases [77]. Although the P element is uniformly distributed on the oxide coating, the XRD does not show any P-containing phase, which indicates that Ca and P-containing compounds are amorphous [77]. On the other hand, for a higher applied potential (≥500 V), new peaks associated with Ca_3_(PO_4_)_2_ and hexagonal HAp (the predominant phase) are observed (Figure 11) [23,55]. These results reveal that the MAO process can successfully produce a crystalline HAp layer on Ta without any pre and post-treatment [55]. The MAO time only influences the intensity of the phase diffraction peaks; no new other phase is generated [65].

The presence of diffraction lines related to HAp, independent of the MAO applied potential, appears after hydrothermal treatment at 140 °C for 24 h [70]. These results are corroborated by XPS analysis, attributing the Ca 2p peaks at 347.1 eV to CaTa_2_O_6_ and 350.7 eV to Ca_3_(PO_4_)_2_. The P 2p peak at 133.3 eV confirms the Ca_3_(PO_4_)_2_ [65,69,70,73]. Still, after a surface annealing at 800 °C for 3 h, a newly formed crystal phase CaTa_4_O_11_ arises [69] and any phase related to HAp appears. Wang et al. [65,69,70] revealed that Ca is mostly compounded into CaTa_2_O_6_ and a residual Ca compound with P to form a small amount of Ca_3_(PO_4_)_2_. In contrast, using an electrolyte only composed of CaA, an orthorhombic CaTa_2_O_6_ phase is noticed, but it is not further detected when β-GP and MgA are added, meaning that Ca is probably bound to them [73].

When the MAO treatment is carried out at ≥350 V [55,65,69,70,71], the formation of calcium tantalate can be described by the reactions of Ca^2+^ ions with the amorphous Ta_2_O_5_ previously developed by the substrate oxidation under a high temperature generated by the micro-arcs [55]; Equation (4).

(4) $${\mathrm{Ta}}_{2}O_{5}+{\mathrm{Ca}}^{2+}++O^{2-}\to {\mathrm{CaTa}}_{2}O_{6}$$

During the annealing treatment, when the temperature is high enough, a great amount of Ta_2_O_5_ is immediately crystallized and then reacts with Ca^2+^ and O^2−^ in the anodic coating, and CaTa_4_O_11_ is formed; Equation (5) [69].

(5) $$2{\mathrm{Ta}}_{2}O_{5}+{\mathrm{Ca}}^{2+}++O^{2-}\to {\mathrm{CaTa}}_{4}O_{11}$$

After the MAO treatment is performed at higher voltages (≥450 V), during the annealing treatment, the amorphous phase converts to crystalline Ta_2_O_5_ and then reacts directly with CaTa_2_O_6_; Equation (6) [69].

(6) $${\mathrm{Ta}}_{2}O_{5}+{\mathrm{CaTa}}_{2}O_{6}\to {\mathrm{CaTa}}_{4}O_{11}$$

**Figure 17:**
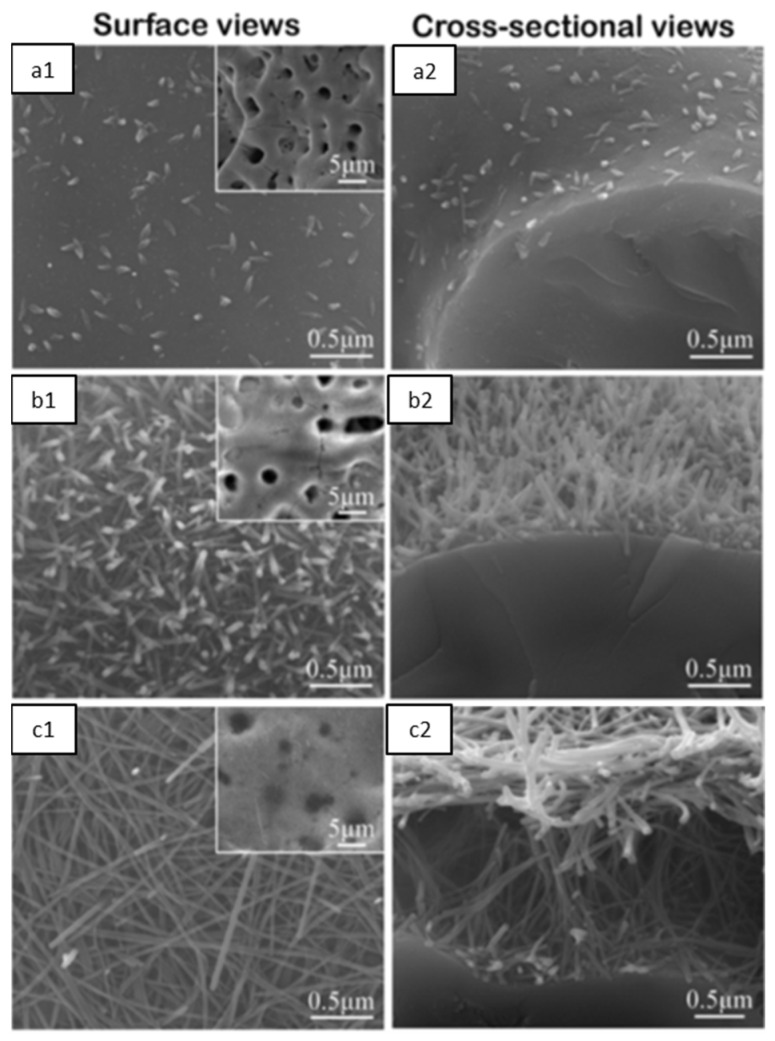
(**a1**–**c1**) Surface and (**a2**–**c2**) cross-sectional SEM images of the outermost parts of the internal walls of macropore, HT for (**a1**,**a2**) 0.5, (**b1**,**b2**) 3, (**c1**,**c2**) 24 h, respectively. Adapted with permission from Ref. [70]. Copyright 2015 Royal Society of Chemistry.

As mentioned, the heat treatment (HT) of MAO-modified Ta surfaces induces the formation of HAp. After 0.5 h of HT, short nanorods, containing Ca and P, are nucleated into the porous coating (Figure 17(a1,a2)). When increasing the HT duration, the nanorods became larger and more evident, covering the micro-porous surface (Figure 17(b1,b2)) and, after 24 h, the nanorods turn into parallel HAp nanofibers (Figure 17(c1,c2)). These results might be explained by the migration tendency of the incorporated Ca^2+^ and PO_4_^3−^ ions out of the oxide coating during the HT, since the intensity of the CaTa_2_O_6_ diffraction peaks decreases, whereas the Ta_2_O_5_ increases. This suggests that a partial conversion occurs due to the Ca^2+^ loss, and CaTa_2_O_6_ turns into Ta_2_O_5_. The Ca^2+^ ions will then react with the PO_4_^3−^ ions present on the surface, forming calcium phosphates. Consequently, the HAp diffraction peaks start to appear. In addition, it is possible to observe that the MAO-treated surface has the highest ions concentration release, which decreases with HT time as more HAp is formed (which has a low solubility) and fewer ions are in the oxide coating [70].

It is interesting to note that the MAO process using an electrolyte of Ca(H_2_PO_2_)_2_ at 500 V leads to the formation of an amorphous oxide coating, but when the Ca(HCOO)_2_ is added to the electrolyte, several new peaks arise that are ascribed to Ca_3_(PO_4_)_2_, Ca_5_(PO_4_)_3_(OH), and CaTa_2_O_6_. On the other hand, when MgA is introduced into the electrolyte, the peaks become assigned to Ca(PO_3_)_2_·2H_2_O, Ca_2_P_2_O_7_, Ca_4_Mg_5_(PO_4_)_6_, and MgCO_3_, and, thus, the surface crystallinity is modified [23]. The presence of phosphates and carbonates is corroborated by the XPS results, although the authors [23] do not justify the presence of nitrogen (N) and silicon (Si) in the survey.

Using Ca- and P-free electrolytes, both Ta and Ta_2_O_5_ phases also appear on porous Ta surfaces [62,75]. Similar results are observed on the X-ray diffraction of non-porous MAO-treated Ta surfaces [67].

### 4.2. Functional Properties of MAO-Treated Ta Surface

#### 4.2.1. Surface Bioactivity and Biocompatibility

As cellular responses are related to the surfaces’ properties [52], MAO-treated Ta surfaces have been widely developed to be used in biomedical devices, tailoring its surface to manipulate both bioactivity and biological responses.

**Figure 18:**
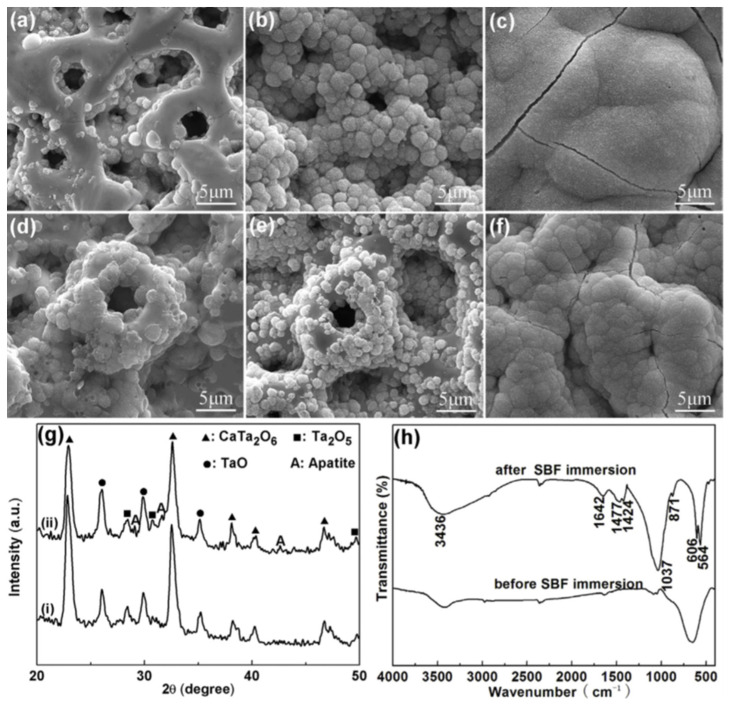
Surface morphologies of Ta_2_O_5_ modified by MAO at 450 V after immersion in SBF for (**a**) 32, (**b**) 36, and (**c**) 40 days; surface morphologies of the Ta_2_O_5_ modified by MAO annealed at 800 °C formed at 450 V after immersion in SBF for (**d**) 9, (**e**) 12, and (**f**) 18 days; (**g**) XRD patterns of the MAO-treated surface formed at 450 V before (i) and after (ii) immersion in SBF for 40 days; (**h**) FTIR spectra of the MAO-treated surface formed at 450 V before and after immersion in SBF for 40 days. Reproduced with permission from Ref. [69]. Copyright 2016 Elsevier.

The surface properties of tantalum oxide, such as surface morphology, crystallinity, oxygen content, and surface wettability, have a strong impact on the surface bioactivity (as a bioactive surface can promote the formation of new apatite) [21,52]. Concerning this, porous CaP-enriched MAO-treated Ta surfaces at 350 and 450 V were characterized regarding their apatite-inducing ability by the bioactivity assay, which consists of the sample’s immersion in simulated body fluid (SBF) for different time points [69]. New spherical-like particles are indeed detected after 42 days of immersion on the Ta surface MAO-treated at 350 V. After 48 days these particles cover the whole surface. If the MAO-treated Ta surface is submitted to a post-annealing, the first particles are detected after 24 days, and, after 30 days, they cover the complete surface. In turn, when the applied voltage is increased up to 450 V, the precipitated particles are detected at 32 days and cover the surface at 40 days (Figure 18a–c). The FTIR spectra of this surface before and after SBF immersion confirm the presence of phosphates on the oxide coating, but also demonstrate that this new apatite formed is carbonated (Figure 18h). The XRD patterns show new diffraction peaks ascribed to apatite after immersion in SBF, revealing that the spherical-like particles are assembled of tiny apatite crystals (Figure 18g). After annealing, an immersion of 9 days is enough for the apatite formation (Figure 18d–f) [69]. In a similar bioactivity assay of an MAO-treated Ta surface doped with CaP at 470 V, the spherical particles are observed after 22 days of immersion in SBF, and, after 28 days, the surface is completely covered. Once more, the post-HT accelerates the apatite formation [70].

In summary, the apatite-inducing ability can be greatly improved generally by Ca and P-enriched porous Ta_2_O_5_ coatings, and also due to a more crystalline phase formed [69]. Moreover, a better apatite-inducing ability is attributed to the higher applied voltage (≥450 V) [69,70], performing post-heat treatments, and the presence of a CaTa_2_O_6_-based coating with well-crystallized HAp nanorods/nanofibers (Figure 17) [70], as well as the presence of crystalline CaTa_4_O_11_ [69]. However, some authors [69] describe the atomic arrangement of CaTa_4_O_11_ as a crystal structure as being more suitable for the epitaxial nucleation of apatite crystals, whereas the structure of CaTa_2_O_6_ (200) hardly matches the apatite structure (0004).

Protein adsorption is the first critical step that determines cell adhesion. Only one paper evaluates the amount of total protein adsorbed on the Ta_2_O_5_ coating from the cellular medium (DMEM) after 24 h of culture. The porous CaP-enriched Ta_2_O_5_ surface significantly improves the protein adsorption when compared to untreated Ta, which can even be enhanced with HT post-treatment [70].

**Figure 19:**
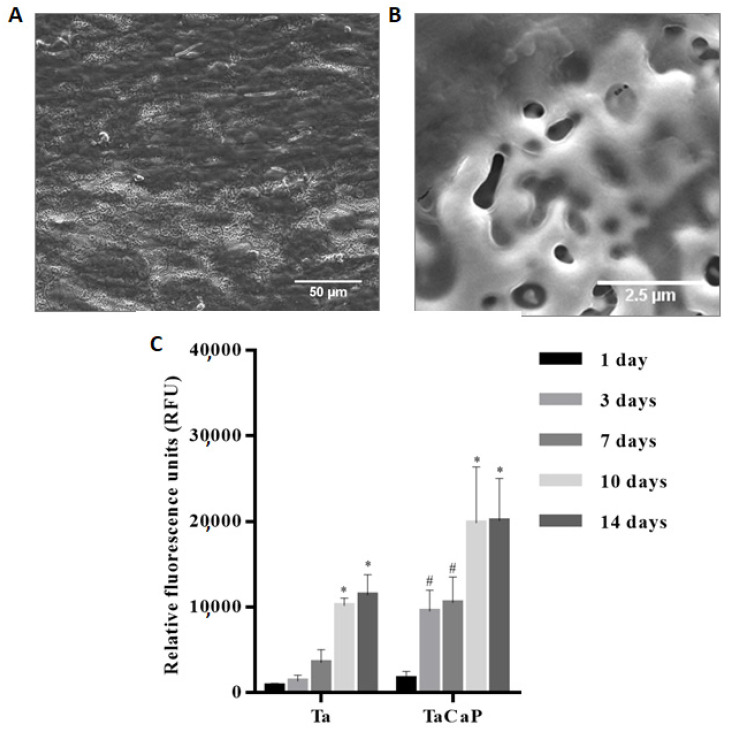
(**A**) Top-view SEM micrographs of MG-63 osteoblastic cells on the MAO-treated Ta surface after culturing for 14 days; (**B**) high magnification image. (**C**) Metabolic activity of MG-63 osteoblastic cells cultured for 14 days over untreated Ta and MAO-treated Ta surface. Significant values as * *p* ≤ 0.05, compared to the initial adhesion on the sample; # *p* ≤ 0.05, compared to the control (Ta surface) for the same time point. Adapted with permission from Ref. [74]. Copyright 2021 Elsevier.

A porous Ta_2_O_5_ surface doped with Ca and P is not toxic for osteoblastic cells, supporting the cells’ adhesion and proliferation [55,70,73,74]. After 14 days of cell culture, Fialho et al. [74] observed that the MG-63 osteoblastic cells fully permeate the porous structure (Figure 19A), showing some thin and long cytoplasmatic extensions in the pores (Figure 19B), as well as significantly promoting the cell viability (translating the initial cell attachment and growth) compared to the untreated Ta surface (Figure 19C). As the cell adhesion to the surface material is mediated by focal adhesions, the vinculin, actin, and nucleus of the osteoblasts on the porous oxide coating are analyzed to demonstrate the cell-binding responses. Well-defined focal adhesions are observed on porous Ta_2_O_5_ surfaces, meaning that the cell adhesion is enhanced by MAO modification [70,73] and, further, HAp post-formation by HT [70]. However, HAp nanofibers resulting from longer durations of HT show a reduced number of focal adhesion and collagen secretion (the main component in the extracellular matrix), inducing cell apoptosis responses, caused by the unstable cell adhesion on the Ta_2_O_5_ surface heat-treated for 24 h [70], which can be related to the surface morphology, as the HAp nanorods become nanofibers, increasing the HT time. Thus, the HAp crystalline phase improves cell adhesion depending on its morphology.

**Figure 20:**
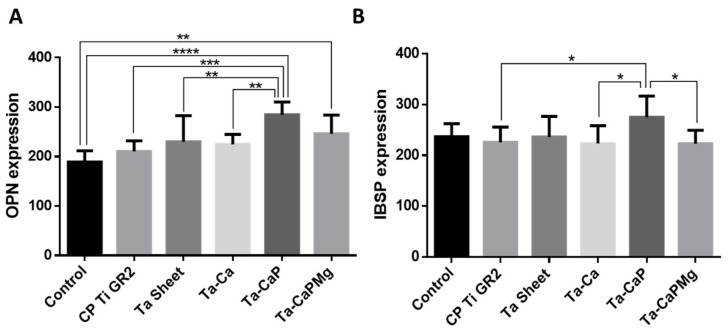
Graphs show (**A**) OPN quantification and (**B**) IBSP quantification represented as mean ± standard deviation. Significant values as **** *p* < 0.0001, *** *p* < 0.001, ** *p* < 0.01, * *p* > 0.05. Adapted with permission from Ref. [73]. Copyright 2021 Elsevier.

Cell differentiation is also improved by CaP-enriched porous Ta_2_O_5_ surfaces. as these surfaces enhance osteopontin (OPN), integrin-binding sialoprotein (IBSP) [73] (Figure 20), and the alkaline phosphatase (ALP) [55] levels in comparison to both the positive control and an untreated-Ta surface. This confirms the surface ability to maintain the cells’ phenotype (inducing the conversion of osteoblasts to bone) [55], thus promoting osteogenesis [73]. Alves et al. [73] studied the influence of the surface properties on the cellular responses. The authors state that the surface properties have a great impact on cell responses, mainly surface chemical composition and wettability, since cell adhesion and gene expressions results are significantly better in the rougher surface with hydrophilic behavior, containing amorphous calcium phosphates, with a higher Ca content on the surface and a Ca/P ratio near the HAp value. They also observe that the surface with the CaTa_2_O_6_ phase (Ca-enriched Ta_2_O_5_ surface) is the MAO-treated Ta surface with a lower focal adhesion area, indicating that this crystalline phase does not promote cell adhesion, which corroborates the explanation reported by Wang et al. [69], which relates the crystalline structure of the predominant phase and the structure of apatite.

Zhao et al. [76] studied the cytotoxicity of the MAO-treated Ta surface using a silicate electrolyte combined with NaOH and under a higher AC potential (450 V). The Si-containing porous Ta_2_O_5_ surface has no cytotoxicity of MG-63 osteoblastic cells, and the integrity of the cells is preserved. The cell proliferation rate of the MAO-treated Ta surface is significantly higher than the untreated Ta surfaces, corroborated by the fluorescence images, where a higher expression of actin microfilaments is observed, which indicates that the porous morphology and the presence of Si promote the osteoblastic growth and spread.

Only one paper of non-porous Ta_2_O_5_ surfaces obtained by MAO investigates the surface bioactivity and the biological responses [67]. After being soaked in SBF for one week, the MAO-treated Ta surfaces display greater spherical-particles precipitation (Figure 15H) than on the untreated Ta surface (Figure 15G). The number of precipitates was significantly higher on the surface submitted to both MAO and NaOH treatments (Figure 15I) [67]. In good agreement, the samples reveal diffraction peaks ascribed to apatite, except for the Ta-untreated surface, maybe due to the small size and low concentration of particles. Besides the strong diffraction lines of Ta, the authors describe the presence of diffraction peaks ascribed to Ta_2_O_5_ and sodium tantalates that are neither represented nor observed in the XRD patterns. Regarding biological characterization, the MTT assay (a colorimetric assay for measuring cell metabolic activity) displays a reduction in viable cells for the MAO-treated surfaces, from 24 h to 72 h of cell culture, revealing some cytotoxic effect, whereas the surface treated by both MAO and NaOH soaking shows biocompatibility. According to the authors, this cytotoxicity can be related to the free ions dissolved from the substrate, or with complex toxic organic species from the scaffold, or with reactive oxygen species (ROS) and reactive nitrogen species involved in cell death. After 24 h of culture, cells show adherent protrusions that are much larger after 72 h and spread over the whole surface, where some calcium crystals are observed. Moreover, the in vivo study shows that both neovascularization and new bone ingrowth occur after 4 and 12 weeks, respectively, confirming the MAO-modified Ta surface as a suitable option for biomedical applications. Although the authors do not present an explanation, the in vitro cytotoxicity can be attenuated in vivo due to the dynamic extracellular fluid flow.

To sum up, CaP-enriched porous Ta_2_O_5_ surfaces reveal no or minimal cytotoxicity to osteoblastic cells, promoting cell adhesion, proliferation, and differentiation when compared to untreated Ta. These cellular responses can be improved with HT or changing the MAO parameters to tailor the chemical composition and surface hydrophilicity. The non-porous Ta_2_O_5_ surface shows some cytotoxicity in vitro but good in vivo outcomes. Regarding these promising outputs, further in vivo studies should be carried out to understand them as a proof-of-concept using both porous (including different porous structures) and non-porous morphologies.

#### 4.2.2. Antimicrobial Activity

**Figure 21:**
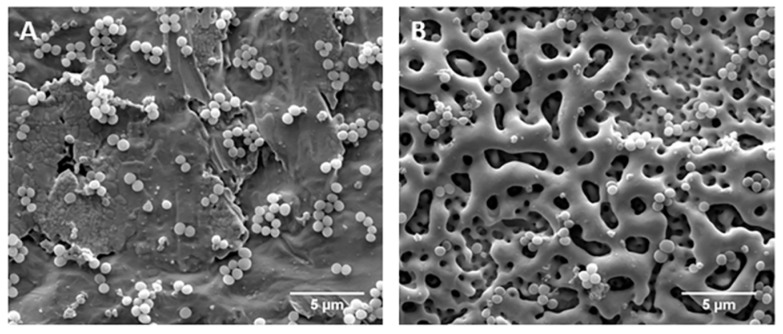
Top view SEM micrographs of *S. aureus* population after 24 h of incubation on (**A**) untreated Ta and (**B**) MAO-treated Ta surfaces. Adapted with permission from Ref. [74]. Copyright 2021 Elsevier.

Up until now, found only two papers were found that investigate the antimicrobial activity of MAO-treated Ta surfaces. Sopata et al. [75] studied the antibacterial activity against *S. aureus* and *P. aeruginosa* bacteria on porous Ta_2_O_5_ surfaces with different surface properties. Both MAO-treated surfaces have no activity against *P. aeruginosa* (Gram-negative bacteria), whereas a growth inhibition zone is observed only in the MAO-treated nanocrystalline Ta surface against *S. aureus* (Gram-positive bacteria), which means that the oxide coating can reduce the bacteria growth and the biofilm formation. This modified Ta surface has an irregular channel morphology with larger pores (Figure 14c,d), and is the thickest, roughest, and most super-hydrophilic surface compared to the MAO-treated microcrystalline Ta (more uniform small pores, thinner and hydrophobic oxide coating) (Figure 14a,b). It is well known that the morphological and physical surface properties have a strong influence on bacteria growth, as well as chemical composition, although the authors do not show any chemical characterization. Using the resazurin assay (a fluorometric/colorimetric assay that indicates the cell viability), Fialho et al. [74] showed that the CaP-enriched porous Ta_2_O_5_ surface does not affect the *S. aureus* viability (Figure 21B) when compared to the untreated Ta surface (Figure 21A), and they demonstrate that the bacteria grow and adhere preferentially inside and around pores, where it is possible to observe an initial biofilm formation. Interestingly, this CaP-enriched porous Ta_2_O_5_ surface has a water contact angle similar to the MAO-treated microcrystalline Ta reported by Sopata, although with a distinct roughness, size pore distribution, and chemical composition.

In this regard, it is fundamental that more studies have to be conducted to understand how the porosity degree, surface roughness, chemical composition, and wettability can influence the bacteria adhesion on MAO-treated Ta surfaces.

#### 4.2.3. Mechanical Properties and Corrosion Resistance

In another area of knowledge, MAO-treated Ta surfaces exhibit interesting properties for mechanical and corrosion applications, which are highly dependent on coating porosity [80,81,82]. A new material implant should be mechanical and corrosion-resistant [13].

Concerning this, the porous Ta_2_O_5_ anodic coating formed by the AC MAO process adheres firmly to the Ta substrate with cohesive strength [65], and shows long-term adhesive strength stability, which decreases with an increase in the applied voltage [69] and HT (attributed to the migration of Ca^2+^ and PO_4_^3−^ ions out of the anodic coating) [69,70]. On the contrary, Sowa et al. [23], who used a DC regime in the MAO process, reported that the adhesion strength is improved with the increasing voltage.

Generally, MAO-treated oxide surfaces have a strong corrosion resistance when compared with metallic surfaces. However, the porous morphology attained by the MAO surface treatment may influence the corrosion resistance of the surface.

In particular, the OCP and potentiodynamic polarization measurements of MAO-treated Ta surfaces under the DC regime demonstrate that the corrosion properties are generally improved with the MAO treatment [62]. However, for an electrolyte composed of K_2_SiO_3_, increasing the applied potential during MAO (from 200 V up to 400 V) does not further improve the Ta corrosion resistance, since the sample that is treated at 200 V has the best corrosion performance, meaning that, under 200 V, the surface features are more compact and denser [62].

**Figure 22:**
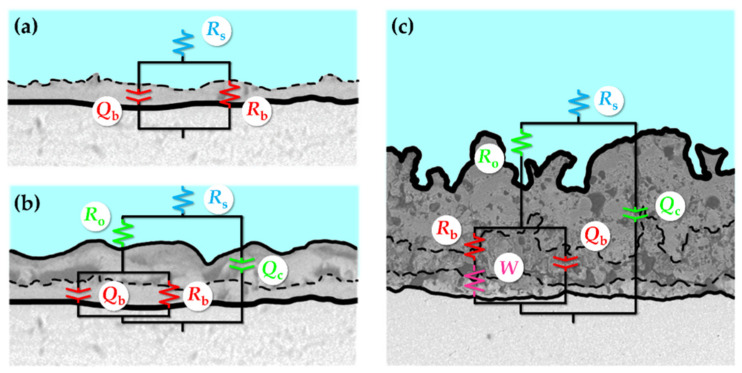
Equivalent electrical circuits used for fitting the impedance spectra of (**a**) the untreated Ta surface; (**b**) the MAO samples that showed two-time constants, and (**c**) those that exhibited two-time constants with a linear constant-phase region. Adapted from Ref. [64].

A more recent paper carries out a deep corrosion resistance investigation of the modified Ta surfaces using different electrolytes mixtures of Ca(H_2_PO_2_)_2_ and Ca(HCOO)_2_ or Mg(CH_3_COO)_2_ combined with an applied potential range from 200 to 400 V (Table 1) [64]. Electrochemical impedance spectroscopy (EIS) is used to determine the total resistance of the biomaterial/electrolyte interface in a corrosion environment (Ringer solution). The results demonstrate that the untreated Ta surface is characterized by a single time constant that can be ascribed with a parallel RC connection (Figure 22a), instead of the MAO-treated Ta surfaces that have at least two-time constants. The RC circuit is based on the resistance of the electrolyte between the reference electrode capillary tip and the sample (Rs) and the parallel resistance of the barrier layer (Rb) and capacitance (Qb) of the passive barrier layer on the flooded untreated Ta surface. Ta surfaces treated by MAO at 200 and 300 V show two well-separated time constants (Figure 22b), where the extra time constant is caused by the presence of a porous layer on top of a dense Ta anodic coating, which is translated by the Ro (electrolyte resistance in the pores of the outer oxide layer) and Qc (total capacitance of the anodic coating) extra pair. For MAO treatments at 400 V, the impedance becomes dependent on the Ta anodic coating composition determined by the MAO electrolyte composition. The electrolytes composed of Ca(H_2_PO_2_)_2_ (electrolyte A) and Ca(HCOO)_2_ (electrolyte B) have more complex spectra inducing Ca and P incorporation, thus exhibiting an almost linear region in the low range of frequencies, suggesting that the ions diffusion phenomenon plays a crucial role in limiting the corrosion rate of treated Ta surfaces. Thus, the correspondent electrochemical circuit has an additional Warburg impedance (W) (Figure 22c), representing the surface elemental diffusion, to improve the fitting of the low-frequency range. The authors conclude that the main factor determining the overall corrosion resistance of MAO-treated Ta surfaces is the polarization resistance of the barrier Ta anodic dense sublayer (Rb), since the best corrosion resistance occurs at low voltages (i.e., 200 V). When the MAO voltage is low enough, the intense sparking is absent, resulting in the development of a thin and porous anodic layer over a bottom dense anodic layer. Independently of the voltage of the MAO process, no evidence of pitting corrosion or oxide breakdown is observed in potentiodynamic polarization measurements. The higher polarization resistance is observed for the more resistive coating, even in more porous coatings.

Sopata et al. [75] also used a Ringer’s solution to test the corrosion behavior of MAO-treated Ta surfaces using an electrolyte composed of a mixture of HF, NH_4_F, glycerin, EG, and H_3_PO_4_. In this case, the influence of the crystallinity of a Ta bare substrate is studied, and, in particular, microcrystalline Ta vs. nanocrystalline Ta before and after MAO treatment. Generally, MAO treatment promotes a corrosion behavior enhancement when compared with the Ta bare substrates. In more detail, although the corrosion resistance between microcrystalline Ta and MAO-treated microcrystalline Ta does not reveal a significant difference, the MAO surface modification over nanocrystalline Ta led to a significant improvement in the corrosion performance. This results from the difference in the grain boundary volume, since a higher volume of grain boundaries into nanocrystalline Ta led to higher electrochemical activity. After MAO treatment, the anodic oxide coating of MAO-treated nanocrystalline Ta significantly inhibits its electrochemical activity and improves the corrosion resistance [75].

In addition, the electrochemical properties of porous MAO-treated Ta surfaces are investigated using a 3.5 wt.% NaCl corrosion environment using an electrolyte composed of Ca(CH_3_COO)_2_·H_2_O and Na_3_PO_4_ [77]. The Nyquist plots show a single capacitive loop, which is influenced by the electric double layer at the interface between the surface and electrolyte. However, the bode-phases’ values of MAO-treated surfaces are slightly lower than the untreated Ta surfaces, indicating that the oxide coatings exhibit relatively poor dielectric properties due to the hierarchical porous structures of the MAO layer, jeopardizing the matrix corrosion resistance. However, at low frequencies, the impedance modulus is more than one order of magnitude higher, meaning that the MAO-treated surfaces have a better corrosion resistance. The potentiodynamic polarization curves display that the untreated Ta surfaces have a passivation current two orders of magnitude higher than MAO-treated surfaces, indicating that the modified surfaces have superior behavior during the corrosion process. The results demonstrate that the double layer of Ta_2_O_5_ provides a positive protection effect for the Ta surface.

Although it is well-known that the anodic coating chemical composition greatly influences the MAO-treated Ta surface corrosion performance, its influence was not studied or reported.

Furthermore, the electrochemical measurements of a non-porous Ta MAO-treated under a bipolar AC constant current density in the absence of anodic sparks indicate that the overpotential for hydrogen evolution has an ohmic dependence on the current density due to the resistance of the anodic coating and electrolyte. The overpotential is enhanced by anodic discharges, which are proposed to be due to the oxygen gas generated during anodic discharges that impede ionic migration during subsequent cathodic polarization [63].

In summary, the MAO process leads to the formation of a protective Ta_2_O_5_ coating, inhibiting the surface corrosion, which has a positive effect on preventing metallic ions release for biomedical applications.

## 5. Conclusions and Further Perspectives

**Figure 23:**
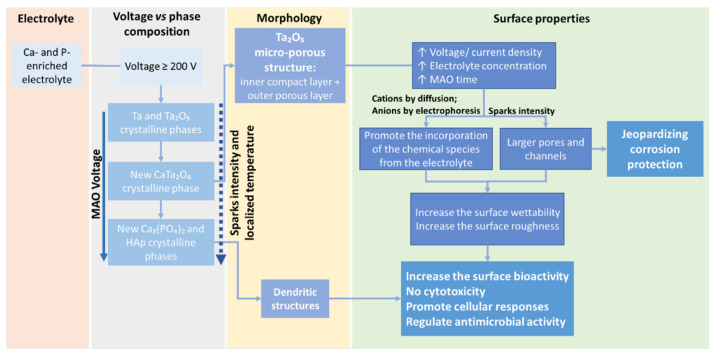
Surface properties of porous Ta_2_O_5_ related to the MAO parameters.

**Figure 24:**
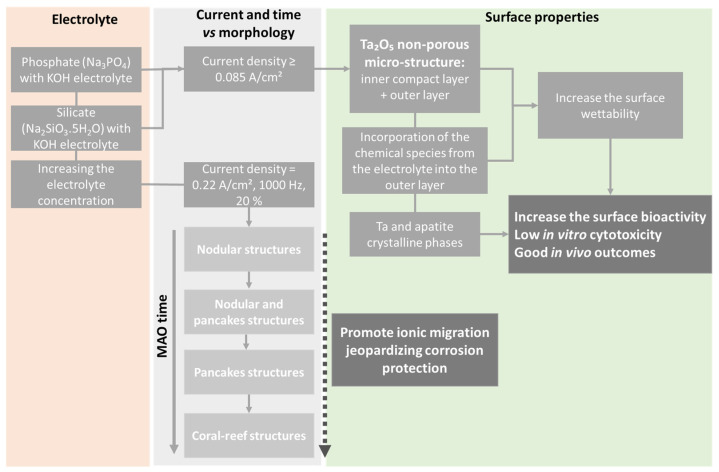
Surface properties of non-porous Ta_2_O_5_ related to the MAO parameters.

Micro-arc oxidation (MAO) is effectively a complex process that embraces different reactions at the same time, as well as different stages, initiated by micro-discharges and plasma formation, followed by gas bubbles evolution, and culminating in the micro-structures formation. Simultaneously, the oxide formation mechanism starts with the oxide coating growing at both Ta/(oxide and oxide/)electrolyte interfaces, which results from the migration of oxygen and tantalum ionic species, along with the oxide coating. The surface morphology and chemical composition development can be tailored by the applied potential/current density and/or electrolyte composition changes. Ta surfaces modified by MAO are reported in a small number of papers, but, globally, all of them agree on the surface properties achieved according to the anodizing parameters, as summarized in Figure 23 and Figure 24.

Most of the papers report that Ta_2_O_5_ microstructures developed by MAO have a very good perspective for biological applications. A Ca- and P-enriched electrolyte (e.g., 0.2–0.7 M CaA and 0.02–0.12 M β-GP with an applied potential ≥200 V) is mostly used to embed these species on the outer porous layer in order to enhance the biological responses, and mostly shows great adhesion to the Ta substrate (Figure 23). In this specific case, if the applied voltage is high enough (>350 V) or if a thermal treatment is used as post-treatment, crystals of calcium phosphates are detected that, combined with the presence of calcium tantalate, significantly enhance the surface bioactivity and, consequently, the cellular interactions, as protein adsorption and the osteoblastic cells’ adhesion, proliferation, and differentiation are enhanced, translating into a quicker healing process and, consequently, leading to the acceleration of osseointegration. Moreover, the reported results show that a porous Ta_2_O_5_ coating promotes a reduction in bacteria growth, and thus the biofilm formation inhibition. This antibacterial activity is enhanced for pore sizes ranging from 0.5 to 2 μm and for channels above 5 μm. Still, similar surfaces show opposite results, addressing some concerns about the antibacterial activity. Although the overall results are promising for the biomedical field, until now, it is still not possible to assert the influence of the surface microstructure (porous vs. non-porous and the different types of porous structures) on both biological responses and antibacterial activity. Through the literature, it is also found that the MAO process (with an applied potential of 200 V) generally leads to the formation of a protective Ta_2_O_5_ coating, inhibiting surface corrosion. However, the MAO coating polarization resistance depends on the chemical composition of the oxide coating and porosity degree, which is greatly influenced by the applied voltage.

Using CaP-free electrolytes (e.g., phosphate and silicate solution), MAO treatment leads to the formation of non-porous Ta_2_O_5_ with two sublayers: an inner compact layer and an outer layer (Figure 24). This outer compact layer can be characterized by the presence of some holes and cracks, which improve the Ta surface bioactivity. When increasing the electrolyte concentration and the applied current density, nodular structures appear in the outer Ta_2_O_5_ coating, which develop towards a mix of nodular and pancakes, and end up as coral-reef features as the MAO time progresses. These morphologic modifications compromise the corrosion protection of the surface.

From a long-term perspective, MAO is primarily used at the industrial scale, and some approaches have been developed to make this technique cost-effective and highly competitive at that scale. However, MAO-treated tantalum surfaces have not been entirely industrialized, mainly due to Ta’s high cost, which makes Ta less profitable.

As a further challenge, a synergetic effort between experimental and simulation studies on the fundamental MAO process over tantalum surfaces should be encouraged, aimed at understanding and developing both high-quality MAO-treated tantalum tailored surfaces and cost-effective solutions for biomedical applications. In particular, it will be interesting to correlate the effect of MAO-treated Ta surfaces with different crystal structures (such as crystalline CaTa_4_O_11_) and morphologies (porous, dendritic structures, nodular, and pancakes) on cellular responses and corrosion protection, as well as in vivo investigations as proof of concept.

The presented results create a playground for designing new micro-scale functional materials with tailored morphologies and chemical compositions in which it is possible to control the surface properties. Hence, from a future perspective, MAO-treated Ta surfaces can be a sustainable alternative to MAO-treated Ti surfaces for high-end products such as electronics, nuclear, naval, aerospace, and automotive industries.

## Data Availability

Not applicable.
